# Domain-specific model selection for structural identification of the Rab5-Rab7 dynamics in endocytosis

**DOI:** 10.1186/s12918-015-0175-x

**Published:** 2015-06-26

**Authors:** Jovan Tanevski, Ljupčo Todorovski, Yannis Kalaidzidis, Sašo Džeroski

**Affiliations:** Jožef Stefan Institute, Jamova cesta 39, Ljubljana, 1000 Slovenia; Jožef Stefan International Postgraduate School, Jamova cesta 39, Ljubljana, 1000 Slovenia; University of Ljubljana, Gosarjeva ulica 5, Ljubljana, 1000 Slovenia; Max Planck Institute of Molecular Cell Biology and Genetics, Pfotenhauer Straße 108, Dresden, 01308 Germany

**Keywords:** Process-based modeling, Dynamical systems, Structural identification, Model selection, Endocytosis

## Abstract

**Background:**

Given its recent rapid development and the central role that modeling plays in the discipline, systems biology clearly needs methods for automated modeling of dynamical systems. Process-based modeling focuses on explanatory models of dynamical systems; it constructs such models from measured time-course data and formalized modeling knowledge. In this paper, we apply process-based modeling to the practically relevant task of modeling the Rab5-Rab7 conversion switch in endocytosis. The task is difficult due to the limited observability of the system variables and the noisy measurements, which pose serious challenges to the process of model selection. To address these issues, we propose a domain-specific model selection criteria that take into account knowledge about the necessary properties of the simulated model behavior.

**Results:**

In a series of modeling experiments, we compare the results of process-based modeling obtained with different model selection criteria. The first is the standard maximum likelihood criterion based solely on least-squares model error. The second one is a parsimony-based criterion that also takes into account model complexity. We also introduce three domain-specific criteria based on domain expert expectations about the simulated behavior of an endocytosis model. According to the first criterion, 90 of the candidate models are indistinguishable. Furthermore, taking into account the complexity of the model does not lead to better model selection. However, the use of domain-specific criteria results in a remarkable improvement over the other two model selection criteria.

**Conclusions:**

We demonstrate the applicability of process-based modeling to the task of modeling the Rab5-Rab7 dynamics in endocytosis. Our experiments show that the domain-specific criteria outperform the standard domain-independent criteria for model selection. We also find that some of the model structures discarded as implausible in previous studies lead to the expected Rab5-Rab7 switch behavior.

**Electronic supplementary material:**

The online version of this article (doi:10.1186/s12918-015-0175-x) contains supplementary material, which is available to authorized users.

## Background

The area of computational systems biology aims at providing computational methods and tools that help in the processes of modeling biological systems, simulating the resulting models, and analyzing their behavior. The modeling process begins with formulating structural hypotheses, i.e., the knowledge-driven identification of the constituent system entities and the interactions between them. There are many modeling formalisms for systems biology that have been developed for the purpose of transformation of structural hypotheses into interpretable and executable models [1, 2]. Since systems biology focuses on dynamical behavior at the molecular level, where change of properties of molecular constituents is observed through time, ordinary differential equations are most commonly used to formulate mathematical models.

In order to refine a conjectured model structure into a complete model, one has to estimate the values of the model parameters. The parameter estimation task is often formulated as a nonlinear optimization problem [3, 4], where the aim is to minimize the discrepancy between the model simulation and the measured behavior of the observed system. Many of the commonly used systems biology tools, such as COPASI [5], CellDesigner [6] and others, focus on the parameter estimation task, considering a single model structure provided by the human modeler.

Recently, computational methods for automated modeling that address both structure identification and parameter estimation, have emerged. On the one hand, probabilistic methods [7] are intrinsically slow and inefficient when applied to large classes of complex model structures. On the other hand machine learning methods for equation discovery [8] are applicable in complex modeling scenarios [9]. A notable recent development is process-based modeling that allows for the integration of knowledge and measured data into the process of inducing mathematical models of dynamical systems [10–12]. These approaches have already been successfully applied in systems biology [13, 14] to the tasks of modeling the structure and dynamics of biological networks from time-course measurement data.

In this paper, we apply process-based modeling to endocytosis, an indispensable part of the cell immune response. Endocytosis is the target of many modeling efforts in systems biology. Del Conte-Zerial et al. [15] present such an effort focusing on the early phase of endocytosis, i.e., the conversion of Rab5 domain proteins to Rab7 domain proteins. They consider a number of alternative model structures, perform careful and extensive comparative analysis thereof and propose a particular cut-out switch structure as the most appropriate model of the Rab5-Rab7 conversion. In a follow-up paper, Tashkova et al. [16] address the task of estimating the parameters in this single cut-out switch model structure.

### Process-based modeling

Process-based modeling is concerned with inducing explanatory models of dynamical systems from data (measurements of the behavior of the observed system) and knowledge (about modeling systems from the given domain). A process-based model describes a dynamical system at two levels of abstraction. At the higher abstraction level, the model is cast as a set of entities (that correspond to system variables) and processes (i.e. interactions between the entities). At the lower abstraction level, each process includes a set of differential and/or algebraic equations, which models the corresponding interaction between the entities involved in the process. While the higher level bears the explanatory power of a process-based model, revealing the structure of its interactions, the lower-level allows for automatic transformation of the model into a set of differential equations that can be used to simulate the dynamical behavior of the observed system.

ProBMoT [17] is a recent implementation of the process-based paradigm for automated modeling of dynamical systems from knowledge and data. It is implemented in Java.

It is still under active development, with the most recent version available for download at http://probmot.ijs.si.

**Fig. 1:**
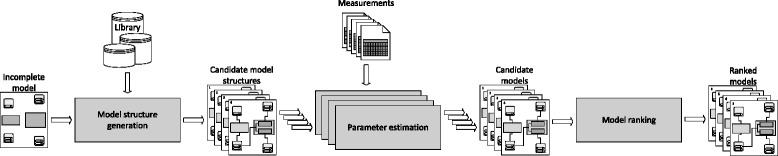
The process of automated modeling with ProBMoT

A graphical description of the process of automated modeling using ProBMoT is presented in Fig. 1. ProBMoT takes as input time-series data, i.e., **measurements** of the dynamical behavior of the observed system. It also takes as input modeling knowledge about the studied domain, represented as a **library** of template model components, i.e., entities and processes. Finally, it takes as input a set of constraints, i.e., an **incomplete model**, that correspond to the particular modeling assumptions made for the specific task at hand.

The library of domain knowledge is a collection of template entities and processes that represent generic components for building models of dynamical systems in the domain of interest. For a particular modeling task, the user specifies an incomplete model that includes a set of entities in the observed system and constraints on the possible interactions between them. The specific entities in the modeling task are instances of the generic template entities in the library. Using them, ProBMoT can enumerate all possible instances of process templates in the library. Following the constraints from the incomplete model, ProBMoT combines these process instances into candidate model structures.

For each candidate model structure, parameter estimation is performed to obtain a set of point estimates of the unknown model parameters that most adequately explains the observed system behavior. To achieve this, parameter estimation in ProBMoT minimizes an objective function that measures the difference between the observed and the simulated behavior. To this end, ProBMoT employs various meta-heuristic optimization methods from the jMetal framework [18] and the SUNDIALS suite for simulating ordinary differential equations [19]. The output of the parameter estimation task represents a candidate model. After the parameter values for all candidate model structures have been estimated, the resulting candidate models are ranked by the minimized value of the objective function. Finally, the ranked list of candidate models represents the output of ProBMoT.

### Model selection

Given the output of ProBMoT, i.e. a ranked list of candidate models, we face the model selection problem of selecting the most appropriate candidate model. By default, the top-ranked model is selected that corresponds to the maximum likelihood criterion for model selection; it only takes into account the least-squares fit to the observed data. However, for the task at hand, the limited observability of protein concentrations and the noise in the measurements pose serious challenges to this default model selection method [16, 20], and the selected model often overfits the observed data.

To address this problem, various model selection criteria have been considered in systems biology [7]. Many of them follow the parsimony principle by combining the least-squares model error with the complexity of the model structure. In addition to this general criterion for model selection, we consider here domain-specific criteria that take into account the expected and necessary properties of model simulations in the particular context of endocytosis. We conjecture that these task-specific criteria for model selection will outperform the other two, general criteria.

Note that the model selection problem is especially important in the context of automated modeling, where large classes of candidate models are being considered. Few computational tools that address the structure identification task (e.g., ABC-SysBio) recast model selection into a parameter estimation task [21]. However, this reformulation requires the user to specify a list of candidate models, a demanding and tedious task for a human modeler. In contrast, process-based modeling offers a more flexible formalism for specifying complex spaces of candidate model structures. Additionally, ProBMoT can consider arbitrary objective functions that correspond to various model selection criteria.

## Methods

First, we are going to cast the task of modeling the Rab5-Rab7 conversion in endocytosis as a process-based modeling task. Then, we are going to formally define the three model section criteria used in the study. Finally, we are going to introduce the experimental setup used for the empirical evaluation and the performance comparison of the models obtained using different model selection criteria.

### Process-based modeling of endocytosis

Process-based modeling formalizes domain-specific knowledge describing entities, that correspond to the variables of the dynamic systems in the domain at hand, and processes, that correspond to interactions between entities. In the particular context of modeling endocytosis, entities correspond to protein domains and processes refer to biochemical interactions between them. The structure of the library is based on a modular formulation of the system of differential equations for modeling the conversion between the Rab5 and Rab7 protein domains [15] of the form:

(1) $$ \begin{aligned} \frac{dr_{5}}{dt} &= K_{1} - (k_{1} + GEF_{5}(R_{5},R_{7}))\cdot r_{5} + GAP_{5}(R_{5},R_{7})\cdot R_{5}\\ \frac{dR_{5}}{dt} &= GEF_{5}(R_{5},R_{7})\cdot r_{5} - GAP_{5}(R_{5},R_{7})\cdot R_{5}\\ \frac{dr_{7}}{dt} &= K_{2} - (k_{2} + GEF_{7}(R_{5},R_{7}))\cdot r_{7} + GAP_{7}(R_{5},R_{7})\cdot R_{7}\\ \frac{dR_{7}}{dt} &= GEF_{7}(R_{5},R_{7})\cdot r_{7} - GAP_{7}(R_{5},R_{7})\cdot R_{7} \end{aligned}  $$

where the variables *r*_5_ and *r*_7_ represent the concentrations of GDP-bound (passive state) Rab5 and Rab7 domain proteins, while *R*_5_ and *R*_7_ represent the concentrations of GTP-bound (active state) proteins. Furthermore, the parameters *K*_*i*_ and *k*_*i*_ represent GDP Dissociation Inhibitor (GDI) association rates and GDI dissociation fluxes respectively. The Rab5-Rab7 interactions labeled with GEF represent activating reactions which catalyze the GDP/GTP exchange by guanine nucleotide exchange factors, while the GAP interactions represent reactions which catalyse the GTP hydrolysis by means of GTPase-activating proteins. The rates of both (GEF and GAP) interactions depend on (are functions of) the GTP-bound state concentrations of Rab5 and Rab7.

**Fig. 2:**
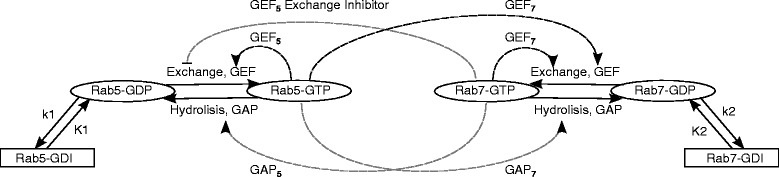
A graphical representation of the Rab5-Rab7 interaction model structure as considered by del Conte-Zerial et al. [15]

Figure 2 provides a graphical representation of the model structure [15], where the dashed lines represent optional interactions between the Rab5 and Rab7 protein domains, while the solid lines represent non-optional (mandatory) interactions. The pointed arrows represent the catalisation (activation) of the corresponding exchange or hydrolysis, while the inhibition of the GDP/GTP exchange by the GEF_5_Exchange Inhibitor is represented by a truncated line. del Conte-Zerial et al. [15] consider a different set of functional forms for modeling each of the four (GEF and GAP) interactions; the combinations of the different functional forms they consider result in only 54 different model structures from the possible 126.

Based on the model structure, we start the development of the process-based library for modeling endocytosis by encoding a single template entity, presented in Table 1, that refers to a general protein domain. The first two variables in the template represent the concentrations of the active-state (GTP_bound_state) and passive-state (GDP_bound_state) proteins. The template includes declarations of two constant parameters that correspond to the dissociation flux and the association rate of the protein molecules in the domain. Note that the template entity from Table 1 represents an arbitrary protein domain. In the particular model of endocytosis from Eq. 1, the template entity instantiates into the two specific entities of Rab5 and Rab7. In the process-based formalism, the variable Rab5.GDP_bound_state_conc, i.e., the GDP_bound_state_conc of the entity Rab5, corresponds to the model variable *r*_5_. Similarly, Rab7.GDI_dissociation_flux corresponds to the model constant parameter *k*_2_.

**Table 1:** Part of the developed library of domain knowledge. Definition of the template entity Protein

**Fig. 3:**
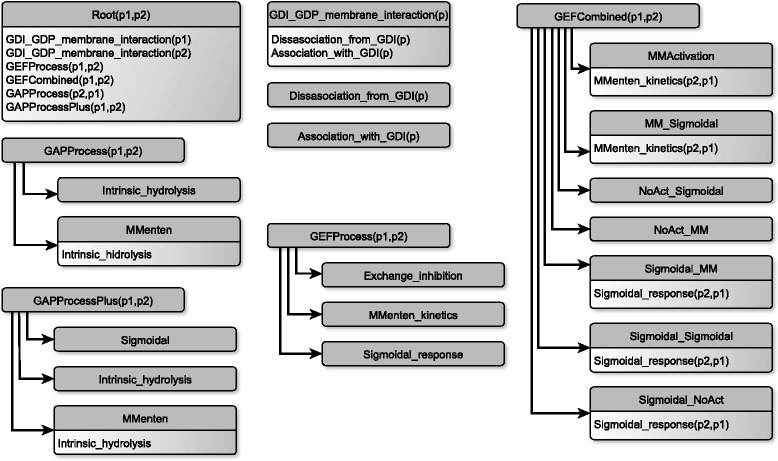
A schematic representation of the process hierarchy in the library. The label of each node denotes the process name, each line in the content of the node denotes a subprocesses

When it comes to the process templates, the ProBMoT library, depicted in Fig. 3, closely follows the general structure of the endocytosis model from Fig. 2. Each node in Fig. 3 corresponds to a template process, where the top node label denotes the template name, while the following lines within the node correspond to subprocesses. The template process Root specifies the way that the models of individual subprocesses are being combined into the system of differential equations presented in Eq. 1.

The hierarchy of process templates specifies the mutually exclusive alternatives for modeling individual subprocesses in terms of the functional forms of the kinetic laws that govern the observed interaction. For example, the process template GDI_GDP_membrane_interaction refers to the interactions between the protein domains and GDI; it contains two subprocesses of Association_with_GDI and Disassociation_with_GDI. The two corresponding process templates specify the specific mass action kinetic laws used in the model. While each of these two process templates specifies a single kinetic law, the two process templates of GAPProcess and GAPProcessPlus specify **two** and **three** alternatives for modeling the hydrolysis of GAP_7_ and GAP_5_ respectively. These include an Intrinsic_Hydrolysis process which models a simple non-catalyzed hydrolysis from the active to the passive state of the protein domain and a Michaelis_Menten process in which the active state of the opposing protein catalyzes the hydrolysis, a process described by a Micaelis-Menten rate. The GAPProcessPlus defines an additional alternative in the form of a Sigmoidal process which describes the catalysis using a kinetic rate following a sigmoidal function.

Similarly, the GEFProcess template describes the **three** alternatives for modeling the GEF_5_ interaction. Two of them describe the auto-catalysis of the exchange, while the Exchange_Inhibition process describes an alternative of the interaction where the second protein inhibits the exchange. Table 2 presents a snippet from a process-based library for modeling endocytosis that illustrates the formalization of individual process templates. It contains specifications of the three mutually exclusive modeling choices for the *GEF*_5_ interaction (i.e., the GEFProcess process template).

**Table 2:** Part of the developed library of domain knowledge. Definition of interaction processes with alternative forms

Finally, the GEFCombined template describes the **seven** alternatives for modeling the GEF_7_ interaction. Note that this process has two components (represented by two arrows in Fig. 2): auto-activation and activation by the second protein, both of which catalyze the exchange between the active and the passive state of the concerned protein. Therefore, some of the alternatives contain subprocesses which account for the auto-catalysis component and have the same functional forms as the auto-activating alternatives for the GEFProcess template.

The whole process-based library for modeling endocytosis, the incomplete model and the task description that have been used to perform all the modeling experiments elaborated later in the empirical part of the paper is available in Additional file 2. Given the library of domain knowledge, ProBMoT enumerates 126 candidate model structures for the particular endocytosis model of interaction between the two protein domains of Rab5 and Rab7. These 126 model structures correspond to the combinations of modeling alternatives specified in the library; the library specifies 2, 3, 3 and 7 alternatives for the four subprocesses of GAPProcess, GAPProcessPlus, GEFProcess and GEFCombined respectively, leading to 2·3·3·7=126 combinations. Note that the candidate model structures considered by ProBMoT include the 54 structures analyzed by del Conte-Zerial et al. [15]. In addition, ProBMoT considered 72 model structures that the authors of [15] dismissed in their manual modeling experiment as trivial and/or structurally flawed. In our automated modeling experiment, we decided to minimize the apriori modeling assumptions and consider all 126 model structures as valid alternatives. The distribution of structure components in all 126 models can be seen in Additional file 1: Figure S1.

Note finally, that the ranges specifying possible values of the model parameters in the library closely follow the ones used in previous studies: [0,2] for the initial values of the system variables (*R*5, *R*7, *r*5 and *r*7), [5,195] for the parameter td in the root process template, and [10^−3^,4] for all the other model parameters. We used the same parameter estimation setting as the ones found to be most suited to the endocytosis modeling task by Tashkova et al. [16], i.e., the optimization method of Differential Evolution [22] with population size of 81, strategy rand/1/bin, differential weight (*F*) of 0.942 and crossover probability (*Cr*) of 0.915. The limit on the number of evaluations of the objective function is 20 thousand times the number of parameters, which amounts to about half a million of evaluations per model structure.

### Model selection

The **standard approach** to parameter estimation is the one of least-squares, where we look for values of the constant parameters that minimize the sum of squared errors between the simulated model output and the observed system behavior. In other words, we minimize a function based on the sum of squared errors, which in the particular case of modeling endocytosis is calculated as the average relative root mean squared error over the two observed variables

(2) $$ {} \begin{aligned} E(m) &= \frac{1}{2} \cdot \left(\sqrt{\frac{\sum_{i} (Rab_{5,i} - \widehat{Rab}_{5,i})^{2}}{\sum_{i} (Rab_{5,i} - \overline{Rab}_{5})^{2}}} \right.\\ &\left.+\sqrt{\frac{\sum_{i} (Rab_{7,i} - \widehat{Rab}_{7,i})^{2}}{\sum_{i} (Rab_{7,i} - \overline{Rab}_{7})^{2}} }\right) \,  \end{aligned}  $$

where *Rab*_5,*i*_,*Rab*_7,*i*_ and $ \widehat {Rab}_{5,i}, \widehat {Rab}_{7,i} $ denote the measured and simulated (using the model *m*) total concentrations of the corresponding Rab domain proteins at the *i*-th time point, while $ \overline {Rab}_{5}, \overline {Rab}_{7} $ denote the mean measured values of the corresponding concentrations across all time points. The *E* measure normalizes the root mean squared error, so that the value of 1 corresponds to the error of a simple baseline model predicting the same mean measured value of the output at each time point.

Note, however, that a sum of squared errors based criterion might not be appropriate for use as a model selection criterion for two main reasons. One is the limited observability of the system variables, which does not provide enough information to discriminate among the different model structures in the space of model structures. The other reason is the risk of over-fitting the noisy data. To address these two issues, we employ three additional model selection criteria.

The following two are **domain-dependent criterion** that take into account the desired behavior of the two system variables that correspond to the concentrations of the active-state Rab domain proteins in the endocytosis model. Namely, when modeling endocytosis, the models of cargo transport through conversion from Rab5 to Rab7 [15, 23] show that the dynamics of the system is controlled by the active, GTP-bound state of the Rab domain proteins, while the concentration of their inactive GDP-bound state remains primarily constant throughout the conversion. Therefore, one would expect that the simulated concentration of the active-state Rab proteins should be highly correlated to the corresponding model output of total (active– and passive-state) protein concentration. Given this expectation about the simulated model behavior, one possible approach is to fit the concentrations of the active-state Rab proteins against the data on total concentrations. This approach was used as an analysis tool for the visual inspection of the model simulation against observed behavior by del Conte-Zerial et al. [15]. However, Tashkova et al. [16] show that this approach fails for parameter estimation, leads to over-fitting of the model to the measured data, and poorly explains the true behavior of the passive state Rab proteins. Here, we first propose an alternative criterion for model selection that discriminates models based on the correlation between the simulated values of the active-state Rab concentrations ($ \widehat {R}_{5}, \widehat {R}_{7} $) with the observed total concentrations (*Rab*5 and *Rab*7). In particular, we measure

(3) $$ \begin{aligned} {}R(m) &= \frac{1}{2} \cdot (\min(1 - r(\widehat{R}_{5}, Rab5), 1) \\ &\,\,+ \min(1 - r(\widehat{R}_{7}, Rab7), 1)),  \end{aligned}  $$

where *r*(*X*,*Y*) denotes the Pearson’s correlation coefficient between the time-series *X* and *Y*. The *R* measure takes values in the range [0,1]. The value indicates the degree of fit to the desired behavior of the hidden system variables, where lower values indicate better correlation between active-state and total protein concentrations.

Based on the same assumption, we introduce a second domain-dependent criterion based on the time when the switch between concentrations of *Rab*5 and *Rab*7 occurs:

(4) $$ X(m) = \frac{\lvert t_{s} - \widehat{t_{s}}\rvert}{t_{max} - t_{0}}  $$

where *t*_*s*_ and $\widehat {t_{s}}$ are the switch time points observed in the measured data and the model simulation respectively, while *t*_0_ and *t*_*max*_ correspond to the first and the last time point. Since we normalize the distance between the switching point in the simulation and in the measured data by the length of the entire observed time interval, the *X* measure takes values in the range [0,1].

We also consider a combination of the domain-dependent criteria *R*(*m*) and *X*(*m*):

(5) $$ RX(m) = \frac{1}{2}\cdot (R(m) + X(m))  $$

To explore the trade-off between the error *E*(*m*) and the different domain-dependent criteria, we introduce the combined criteria,

(6) $$\begin{array}{@{}rcl@{}}  ER(m) = \alpha\cdot E(m) + (1 - \alpha)\cdot R(m), \end{array} $$

(7) $$\begin{array}{@{}rcl@{}}  EX(m) = \alpha\cdot E(m) + (1 - \alpha)\cdot X(m), \end{array} $$

(8) $$\begin{array}{@{}rcl@{}}  ERX(m) = \alpha\cdot E(m) + (1 - \alpha)\cdot RX(m), \end{array} $$

where *α* is a trade-off parameter in the range [0,1]. The value of 0 leads to model selection based purely on the domain-dependent criteria, while the value of 1 leads to model selection based purely on the error *E*(*m*).

Finally, we also consider a general, **domain independent criterion** commonly used to avoid overfitting, based on the parsimony principle. Following this principle, from a number of models with comparable error, we select the simplest one. Model selection approaches that follow the parsimony principle, such as the Akaike information criterion or minimal description length [24], deal with finding a trade-off between the model error and the model complexity. In the particular case of process-based models, we measure the complexity of a model as the number of processes in the model structure, i.e., *C*(*m*)=#processes(*m*). In turn, we introduce the *β* parameter to trade-off between the model error (degree-of-fit) and complexity, as follows:

(9) $$ EC(m) = \beta\cdot E(m) + (1 - \beta)\cdot C(m),   $$

where the value of the trade-off parameter *β* is in the range of [0,1].

A more complicated, domain-dependent, version of this criterion can be derived from equations 6-8 and equation 9, which trade-off between the domain-dependent criterion (instead of the error *E*(*m*)) and the model complexity *C*(*m*)

(10) $$\begin{array}{@{}rcl@{}} ERC(m) = \beta\cdot ER(m) + (1 - \beta)\cdot C(m), \end{array} $$

(11) $$\begin{array}{@{}rcl@{}} EXC(m) = \beta\cdot EX(m) + (1 - \beta)\cdot C(m), \end{array} $$

(12) $$\begin{array}{@{}rcl@{}} ERXC(m) = \beta\cdot ERX(m) + (1 - \beta)\cdot C(m). \end{array} $$

For example, as *ER*(*m*) combines *E*(*m*) and *R*(*m*), *ERC*(*m*) combines *E*(*m*), *R*(*m*), and *C*(*m*). When *α*=1, *R*(*m*) is not taken into account and the *ERC*(*m*) model selection criterion becomes the (domain-independent) trade-off between the model error and complexity, i.e., *EC*(*m*).

### Evaluation of modeling performance

Before we test our central hypothesis that the domain-specific model selection criteria are best suited for modeling endocytosis, we define the metrics that we use to measure the modeling performance of ProBMoT.

**Fig. 4:**
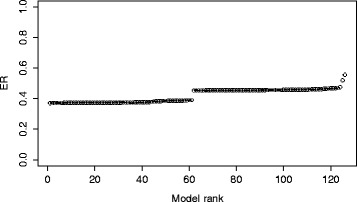
Error profile. Sorted ranking of the 126 models according to the estimated values of the *E* *R* criterion. The trade-off parameter setting is *α*=0.5. Two long and two short plateaus can be identified in this error profile

The first performance metric describes the ability of the model selection method to discriminate between the 126 model structures considered by ProBMoT. To measure the discriminative power of a particular model selection criterion, we run a ProBMoT experiment where the given criterion is used to rank the models. We then depict the error profile, i.e., plot the value of the given criterion for each model against the increasing model rank; see Fig. 4 for an example. Furthermore, we refer to the initial flat region of the error profile as the plateau; its length equals the number of models it contains. A simple heuristic for detecting the plateau is the test whether there is more than 10 % error difference between two consecutive points. The first such difference indicates the end of the plateau. For example, the plateau of the error profile in Fig. 4 contains 62 models. Note that the plateau represents the set of top-ranked model structures that are indistinguishable in terms of the model selection criterion used to rank them. The fewer models in the plateau, the better the performance of the model selection criterion, i.e., its ability to discriminate between the candidate model structures.

Second, we compare the model structures in the first plateau with the three groups of the models that have been identified and grouped by their ability to produce bistable behavior [15]. The first group includes 26 models that can reproduce the bistable switch behavior from Rab5- to Rab7-dominated steady states, some of them follow a toggle switch, others a cut-out switch. We will refer to this first group of models as COT. The second group includes 18 models that follow an in-phase switch; we will refer to this second group of models as IP. The third groups includes 10 models that can not reproduce a bi-stable switch behavior; we will refer to this third group of models as NOBS. For the model structures in the first plateau, we are going to investigate the average rank of the models in each of these three groups as an indicator of the performance of our approach. We expect higher average rank and number of models in the first plateau that belong to the COT and IP groups, and relatively smaller average rank and number of models from the NOBS group. In order to make a fair evaluation of the performance of our approach and the chosen model selection criteria we completely exhaust all previously identified structural possibilities. Since stability analysis has not been performed on the remaining 72 model structures, we consider them to be in a separate fourth group.

Third, we analyze the structure and behavior of the best models. We aim at identifying the structure patterns for the models in the first plateau: to this end, we analyze the frequencies of the different modeling choices in the first-plateau models for the four functions of GEF_5_, GAP_5_, GEF_7_ and GAP_7_. We also report the structure and the simulated behavior of the top-ranked model. We repeat this analysis for the models in the first plateau that also belong to the COT and the IP groups.

Finally, we consider the problem of practical parameter identifiability, i.e. the uniqueness of the estimated parameters for a candidate model given the available measured data. A systematic study of a large number of systems biology models [25] and previous studies of the problem of identification of the model of the Rab5-Rab7 switch in endocytosis [16], indicate identifiability problems: Parameters in models from the area of systems biology are uncertain in general and the model proposed in the original study has specific practical parameter identifiability problems. Nevertheless, we investigate the possibility of further discrimination of the models based on this property, and the possible improvement of the identifiability given the best found combination of domain-dependent and independent criteria for optimization.

We follow the bootstrap method, proposed by Joshiet al. [26], to perform the parameter identifiability analysis, choosing it for several reasons. First, it provides more reliable estimates of the parameter confidence intervals compared to, for example, the Fisher-Information-Matrix based method. Second, it is better suited for highly non-linear models with high parameter-value uncertainties. Third, the same method was used to perform parameter identifiability of an endocitosys model [16]. Note however, that the bootstrap method comes with a high computational cost since it requires a large number of parameter estimations on the same model structure using different data set with added random noise at a certain noise level. The obtained parameter estimates are then used to analyze the distribution of the values of individual parameters and the corresponding confidence intervals. We perform the parameter identifiability for the three selected models: the top-ranked model, the top-ranked COT model, and the top-ranked IP model.

### Ethics approval

No aspect of this study required ethics approval.

## Results

In the experiments, we vary the values of the trade-off parameters *α* and *β* in the range [0,1] with a step of 0.1. For each pair of values, we perform a single modeling experiment by running ProBMoT with the corresponding model selection criterion. We analyze the results of the experiments in terms of the performance metrics presented in the previous section.

### Data

The data set used in the experiments of modeling endocytosis is derived from the measurements used by del Conte-Zerial et al. [15] and is available in Additional file 3. These include measurements collected by tracking early endosomes in three independent experiments that lead to 28 time courses of Rab5 and Rab7 intensity. The data from different experiments and time courses were then aggregated by carefully performed manual scaling and averaging into two time-series of length 10,571 time points along the time interval of [-5, 300] seconds, where the time point 0 corresponds to the Rab5-Rab7 conversion switch point [15]. Finally, to use the same alignment of the data against the model simulation as in previous studies [15, 16], we shifted the time axis using the transformation *t*←*t*+828.56.

Note that, due to the limitation of the measurement equipment, only the total (that is active– and passive-state) concentrations of the Rab5 and Rab7 domain proteins are observed: The observed values at each time point correspond to *Rab*5=*R*5+*r*5 and *Rab*7=*R*7+*r*7, respectively. Recall from Equation (1) that *R*5,*R*7 and *r*5,*r*7 correspond to the concentrations of the active (GTP-bound) and passive (GDP-bound) state of the Rab domain proteins, respectively. To deal with the limited observability of the system variables in the ProBMoT model, we define its outputs as (rab5.GDP_bound_state_conc + rab5.GTP_bound_state_conc) * K and (rab7.GDP_bound_state_conc + rab7.GTP_bound_state_conc) * K, where K denotes a scaling factor that allows for proper matching of the measured data against the simulated model outputs. Note that the range of values of K considered by ProBMoT is [10^3^,10^5^] [16].

### Domain-independent model selection

**Fig. 5:**
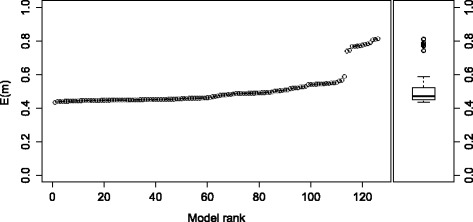
Error profile and a box plot of the error obtained using the criterion *E*. Sorted ranking of the 126 models according to the estimated values of the *E* criterion

We start by analyzing the modeling results obtained with the default ProBMoT selection criterion of *E*, which corresponds to the setting of the trade-off parameters *α*=1, *β*=1. As expected, all model errors are in a very narrow range, shown in the plateau and box-plot in Fig. 5. The plateau of size 113 shows that almost 90 % of all candidate models are indistinguishable in terms of the *E* criterion.

**Fig. 6:**
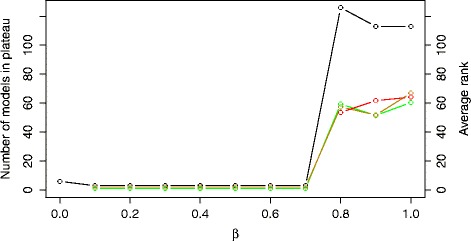
The size of the error-profile plateau (black line) and the average ranks of the structures belonging to the COT (green line), IP (yellow line) and NOBS (red line) group obtained using the criterion *E* *C*. The plot is obtained by varying the values of the *β* trade-off parameter in the range [0,1] with an increment of 0.1

One of the approaches to distinguish between model structures is to perform model selection using both model error and complexity, i.e., using the *EC* model selection criterion. The distribution of the complexity of the models is shown in Additional file 1: Figure S2. Figure 6 shows the influence of the *β* trade-off parameter on the plateau size (black line-points) and the average ranks of the COT (green line-points), IP (yellow line-points) and NOBS models (red line-points). Note that small *β* values lead to short plateaus including only the simplest model structures, i.e., those including six processes, indicating a strong preference towards simple models. The simulated behavior of these models differs significantly from the expected bi-stable switch behavior. On the other hand, high *β* values lead to modeling performance comparable to or worse than the model selection criterion *E*.

### Domain-dependent model selection

In a similar manner, we explore the performance of the *ER*, *EX* and *ERX* model selection criteria that trade-off between model error and model fit to the desired behavior of the hidden system variables. We find that the domain-dependent criteria lead to remarkable improvement in discriminative power over the domain-independent model selection criteria.

**Fig. 7:**
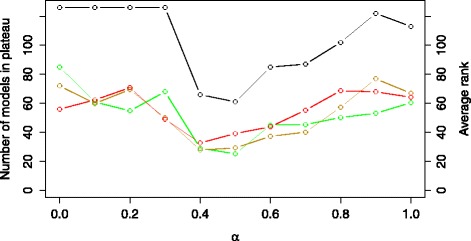
The size of the error-profile plateau (black line) and the average ranks of the structures belonging to the COT (green line), IP (yellow line) and NOBS (red line) group obtained using the criterion *E* *R*. The plot is obtained by varying the values of the *α* trade-off parameter in the range [0,1] with an increment of 0.1

Figure 7 shows the influence of the change of the trade-off parameter *α* on the plateau size and the average ranks of the COT, IP and NOBS models in the list of models ranked using the *ER* criterion. Small and large values of *α* lead to large plateaus, with a significant drop of the plateau size for *α*=0.4 and a minimum at *α*=0.5. Note that this value also leads to the smallest average ranks of the plausible model structures. Additional file 1: Figure S3 provides further details on the results of the modeling experiment using the *ER* criterion with *α*=0.5. The size of the plateau is 62, i.e., less than 50 % of all the candidate models; a significant improvement in discriminative power over the 90 % obtained with *E*. Out of these 62 models, 13 have structures belonging to the COT group, 8 to the IP group, and 6 to the NOBS group. The range of errors is tight with a mean value of 0.42, a median of 0.45 and a standard deviation of 0.04. Note that the obtained behavior of some of the models in the first plateau can be considered as unsatisfactory, for example, the simulation of one of the active-state concentrations of the proteins can be uncorrelated to the corresponding measured density even though the correlation is taken into account within the *ER* criterion during optimization. We believe that this is due to the strong influence of the *E* component in the used criterion, combined with the imperfect optimization and the identifiability issues presented below.

**Fig. 8:**
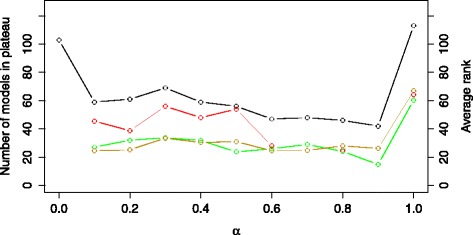
The size of the error-profile plateau (black line) and the average ranks of the structures belonging to the COT (green line), IP (yellow line) and NOBS (red line) group obtained using the criterion *E* *X*. The plot is obtained by varying the values of the *α* trade-off parameter in the range [0,1] with an increment of 0.1

Figure 8 shows the influence of the *α* value on the plateau size and the average ranks of the COT, IP and NOBS models in the list of models ranked using the *EX* criterion. The curve corresponding to the plateau size has a similar saddle-like shape as the one for the *ER* criterion from Fig. 7. The smallest plateau size is obtained for *α*=0.9. The size of the plateau is 42, reducing the percentage of candidate models in the plateau to 33 %, which represents a further improvement over the *ER* criterion. Additional file 1: Figure S4 provides details on the results of the modeling experiment using the *EX* criterion with *α*=0.9. For values of *α*=0.7 and *α*=0.9, we find no structures belonging to the NOBS group in the plateau. In the smallest plateau, out of the 42 models, 15 have structures belonging to the COT group, 11 to the IP group and none to the NOBS group. The range of errors is significantly wider in comparison to the best case using the *ER* criterion with a mean equal to 0.65, a median of 0.73 and a standard deviation of 0.33, which leads to the overall conclusion of significantly improved discriminative power. In contrast to the experiments using the *ER* criterion, the behavior of the models in the plateau, regarding the optimized point of switch, is within the boundaries of the expected, i.e. there is no unsatisfactory behavior.

**Fig. 9:**
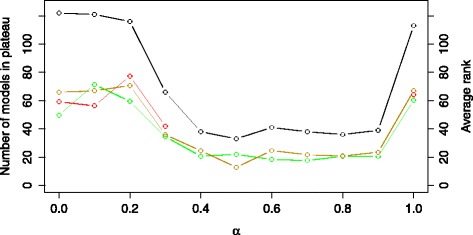
The size of the error-profile plateau (black line) and the average ranks of the structures belonging to the COT (green line), IP (yellow line) and NOBS (red line) group obtained using the criterion *E* *R* *X*. The plot is obtained by varying the values of the *α* trade-off parameter in the range [0,1] with an increment of 0.1

Combining the two domain-dependent criteria brings further improvements. Figure 9 shows the influence of *α* on the plateau size and the distribution of the ranks of the plausible model structures in the plateau using the *ERX* criterion. We observe a smooth saddle like shape of the plateau size as a function of *α*. The smallest plateau size is obtained for *α*=0.5. The size of this plateau is 33, reducing the percentage of candidate models in the plateau down to 26 %. Additional file 1: Figure S5 provides details on the results of the modeling experiment using the *ERX* criterion with *α*=0.5. There are no structures shown to not achieve bistable behavior in the plateau for values of alpha larger than 0.3 and smaller than 1.0. In comparison to using the *EX* criterion, the use of the combined *ERX* criterion leads to a slightly smaller number of models that have been shown to reproduce bistable behavior, slightly tighter range of error values and improved overall quality of the models regarding their fit to the data and the dynamic behavior of the components of the system. In the smallest plateau, out of the 33 models, 10 have structures belonging to the COT group, 7 to the IP group and none to the NOBS group. The range of errors has a mean equal to 0.47, a median of 0.44 and a standard deviation of 0.27. Using the combined criterion, no models in the plateau produce unsatisfactory behavior.

**Fig. 10:**
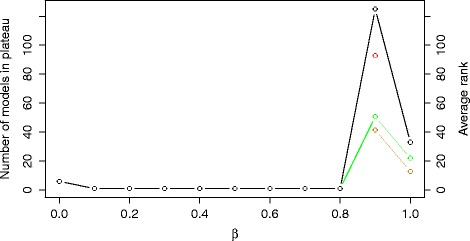
The size of the error-profile plateau (black line) and the average ranks of the structures belonging to the COT (green line), IP (yellow line) and NOBS (red line) group obtained using the criterion *E* *R* *X* *C*. The plot is obtained using the value *α*=0.5 and by varying the values of the *β* trade-off parameter in the range [0,1] with an increment of 0.1

Going one step further, we combined the best performing domain-dependent criterion *ERX* (for *α*=0.4) with the normalized model complexity, to experiment with the combined *ERXC* criterion. Figure 10 shows the results of the experiments with varying values of the trade-off parameter *β*. They are similar to the case of using the *EC* criterion.

For the problem of modeling the Rab5-Rab7 switch in endocytosis, no further improvements of discriminative power can be achieved by considering the complexity of the model structure for model selection. The optimized values for each of the used criteria are uncorrelated to the complexity of the model structures. The distribution of errors for each criterion for each possible complexity of the model structures can be seen in Additional file 1: Figure S6.

Overall, the comparisons of the modeling results obtained using different values of *α* and *β* reveal that the *ERX* modeling criterion with *α*=0.5 has the best ability to discriminate between the candidate model structures.

For completeness of the results, Additional file 1: Table S1 presents the values of all the components of the combined *ERX* criterion, i.e., *E*, *R* and *X* for the 33 models in the first plateau of the *ERX* error profile from Additional file 1: Figure S5. Additional file 1: Table S2 presents the values of all the components of the *ERX* criterion for the least complex and the most complex models.

### Analysis of the obtained models

**Fig. 11:**
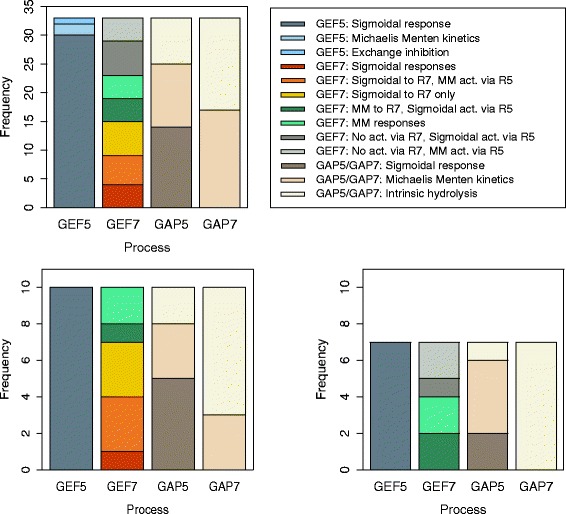
Distribution of the structural components of the models in the plateau using the *ERX* criterion with *α*=0.5. The distribution of the components of all models (top left), the distribution of the components of the models in the plateau belonging to the COT group (bottom left) and the distribution of components belonging to the IP group (bottom right)

We begin the analysis of the best obtained models, i.e., those in the first plateau of size 33, by analyzing the distribution of the components of their structures. The distribution is shown in Fig. 11. For the entire plateau of models, it can be seen that there is a major shift in distribution for the GEF5 functional forms in favor of the Sigmoidal response, which is even more obvious when considering the distribution in the major classes of bistable models in the plateau. A minor shift in distribution is present in the GAP5 functional forms favoring the Sigmoidal response, which can be also observed in the distribution for the major classes of bistable models. In general, the evidence is in favor of positive regulation of the hydrolysis of active-state to passive-state Rab5 via Rab7 as opposed to no regulation (intrinsic hydrolysis).

While there is no significant shift in distribution for the GEF7 and GAP7 functional forms in the entire plateau, there are important differences in the specific groups of models. For the GEF7 process in models of the COT group an auto-catalytic process of exchange must be present. We observe a more frequent Sigmoidal than Michaelis-Menten response to the active-state Rab7. In models of the IP group, the requirement for an auto-catalytic process is not apparent. If it is present, however, it takes the form of a Michaelis-Menten response to the active-state Rab7. It can be also seen that the exchange of passive– to active-state Rab7 is positively regulated by active-state Rab5 in all cases.

For the GAP7 process, for both COT and IP group, the Intrinsic hydrolysis alternative for the GAP7 process is favored. This is indicative of an absence of regulation of the hydrolysis of active-state to passive-state Rab7 via Rab5, which is especially clear in the case of models from the IP group.

We next take a closer look at a sample of three endocytosis models from the plateau. We consider the top-ranked model overall and the best locally ranked models in the first plateau from each class of models.

**Fig. 12:**
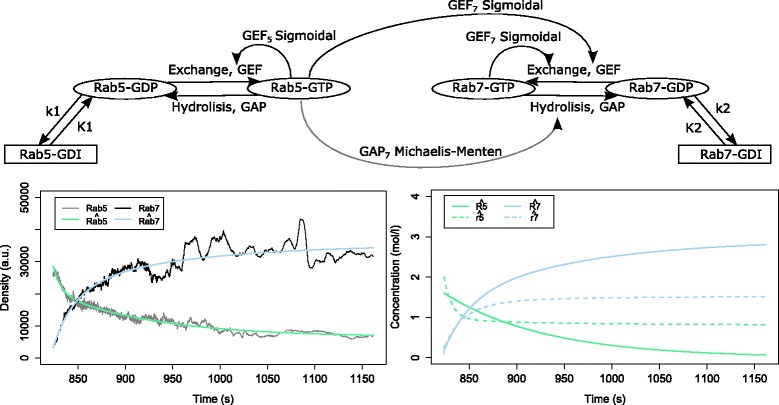
The structure (top), the output behavior (bottom left) and the behavior of the active and passive state protein concentrations (bottom right) of the top-ranked model, obtained using the *ERX* criterion with *α*=0.5. The model has an error *E* *R* *X*=0.126

Figure 12 depicts the structure of the top-ranked model overall, its simulated output behavior compared with the measurements, and the simulated behaviors of the hidden system variables (*R*_5_, *R*_7_, *r*_5_ and *r*_7_) representing the concentrations of the active and passive states of the protein domains. The simulation of the total densities of the protein domains has a reasonable fit to the measured data. The structure of the model leads to a switch behavior due to the strong influence of Rab5. However, there is no feedback mechanism which will allow for transition from one to another stable behavior.

**Fig. 13:**
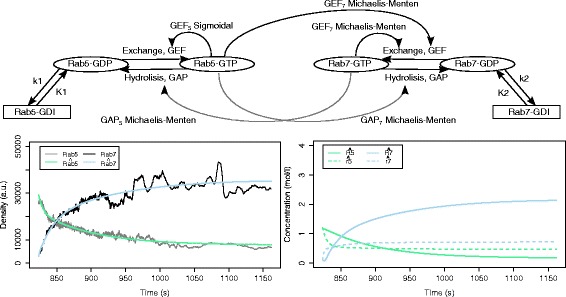
The structure (top), the output behavior (bottom left) and the behavior of the active and passive state protein concentrations (bottom right) of the best ranked model from the COT group, obtained using the *ERX* criterion with *α*=0.5. The model is ranked fourth overall and has an error *E* *R* *X*=0.129

Figure 13 depicts the structure of the top-ranked model having a structure belonging to the COT group. It is ranked as fourth overall. The simulation of the total densities has a good fit to the measured data, both qualitatively and quantitatively indistinguishable from the simulation of the top-ranked model. The simulation of the active and passive components of both protein domains achieve the expected behavior. The dynamics of the active states of the protein domains drives the dynamics of the system and their switching time corresponds to the switching time observed in the measurements. The passive state concentrations remain stable throughout the time of simulation.

The structure of the model allows for a cut-out switch behavior due to the strong positive influence of Rab5 on the exchange of passive to active-state Rab7 combined with auto-activation of the exchange, which overpowers the influence of Rab5 on the hydrolysis of Rab7 on one hand, and the negative feedback from Rab7 to Rab5, which leads to low concentrations of active-state Rab5 on the other.

**Fig. 14:**
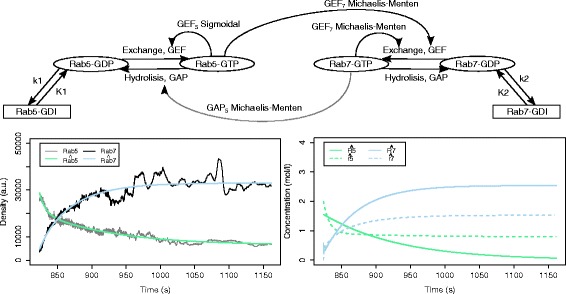
The structure (top), the output behavior (bottom left) and the behavior of the active and passive state protein concentrations (bottom right) of the best ranked model from the IP group, obtained using the *ERX* criterion with *α*=0.5. The model is ranked fifth overall and has an error *E* *R* *X*=0.129

Figure 14 depicts the structure of the top-ranked model having a structure belonging to the IP group. It is ranked fifth overall. As with the previous models, the simulation of the total densities has a good fit to the measured data. The simulation of the active and passive state component concentrations is qualitatively like the one of the previously discussed model. The structure reveals the reason for the similar behavior.

Compared to the best COT model, the best IP model is missing only a *GAP*_7_ interaction. The other present interactions have the same functional forms. The dynamics and the bistable behavior arise from the same sources discussed above, lacking only the negative feedback from the active-state Rab5 via *GAP*_7_.

The practical parameter identifiability analysis performed on the selected model structures shows, as expected, parameter identifiability problems. Although there is a slight improvement in the relative size of the confidence interval to the mean and the estimates for all models, overall the conclusions from our results correspond with the conclusions from previous experiments [16] on a related model.

The summarized statistics of the identifiability analysis for each model can be seen in Additional file 1: Tables S3–S5. The uncertainties (length of the 95 % confidence interval) are large for a significant number of parameters values for all functions, independent of the functional alternative in the selected models.

The shape of the distribution of the parameters differs significantly in most of the cases from the normal distribution, indicating non-linearity of the systems with respect to their corresponding parameter values; see the histograms shown in Additional file 1: Figures S8, S10 and S12. This difference is most evident in the top-ranked model belonging to the IP group in contrast to the shapes of the distributions of parameter values of the top-ranked model structure. For the majority of the parameters, their values were most frequently estimated to be in close proximity to the bounds of the allowed range.

The correlation matrices for all model structures (Additional file 1: Figures S7, S9 and S11) show high absolute correlation values for certain sets of parameters. In all three models, we observe high correlation of the association rate and the dissociation flux of the proteins with GDI.

In the top-ranked model, there is high correlation between the estimated values of the parameters of the auto-catalysis component of GEF7 and between the estimated values of the GAP7 intrinsic hydrolysis rate and the maximum rate parameter in the Michaelis-Menten term. There is a high positive correlation between the estimated initial values of the active and passive states of Rab5 and a high negative correlation between the estimated initial values of the active and passive states ofRab7.

In the top ranked model from the COT group, we observe a high correlation between the intrinsic hydrolysis rate and the maximum rate parameter in the Michaelis-Menten term values in both the GAP5 and the GAP7 functions. As for the top-ranked model, there is a high positive correlation between the estimated initial values of the active and passive states of Rab5 and a high negative correlation between the estimated initial values of the active and passive states of Rab7.

In the top ranked model from the IP group, we observe high correlation between all of the parameters of the GEF5 and GAP5 function. There is a positive correlation between the estimated initial values of the active and passive states of Rab5.

## Discussion

The combination of limited noisy observations, on one hand, and the expectations about the behavior of the unobserved system variables, on the other, poses a difficult model selection problem. We approach this problem by combining several criteria for model selection. Two are the standard model selection criteria of model error and simplicity and three are based on the expected behavior of hidden system variables.

The comparison of different criteria shows that the simplicity-based criterion leads to little or no improvement of discriminative power; the majority of the model structures remain indistinguishable. This is also evident from the low correlation of the optimized values for each of the used criteria and the complexity of the model structures. The plateaus are not a result of over-fitting and cannot be avoided by considering the principle of parsimony. On the other hand, a combination of a domain-independent least-squares based optimization criterion with a simple problem-specific criterion is better suited to the real-world problem at hand than the simplicity-based criterion. In our experiments, the combination of the domain-independent criterion with two different domain-dependent criteria leads to additional improvement. The introduction of domain-specific criteria leads to significantly improved selectivity of the process-based modeling algorithm. In the case of modeling endocytosis, this improvement is evident from the absence of those models which have been previously shown to have monostable behavior (NOBS group), whose average rank (or lack thereof) in the plateau we show in red color in the plots for each criterion.

The simulation of the dynamics of both the observed total density and the unobserved states of the protein domains provides a good fit to the measured data and expected dynamical behavior of the components of the system. This property is consistent in the best ranked models. Due to the existing parameter identifiability problems in all selected representative models, further discrimination (based on the identifiability) cannot be made.

A number of models in the first plateau (even in the experiment using the combination of criteria that has the highest selectivity) do not belong to any of the COT, IP or NOBS groups. Among these, there are some that might be considered as structurally flawed under some expectations for structural mechanisms as is the case with the missing feedback mechanism in the top-ranked model. The presence of these structures may be (in part) a result of overfitting due to the complex representation of processes, the number of free parameters, the limited observability, and the quality of the data. Nevertheless, some of these previously identified (but not considered) models, given their performance, might lead to the reconsideration of parts of or their complete structure in further studies.

We consider the introduction of domain-specific criteria and the performed comparison to be an important step towards improved automated modeling approaches and a solution of the model selection problem. The majority of model selection criteria employed in the domain of systems biology are based either on likelihood, on the Bayesian principle or a combination of the previous [7], due to their well-established reputation in other areas. Most of them have the principle of parsimony implicitly encoded. On the other hand, in biology, the principle of parsimony should be sometimes set aside in favor of selecting better (although more complex) explanatory models [27]. We argue that knowledge-based, domain-specific criteria for model selection should be considered prior to or in conjunction with approaches based on the parsimony principle. These criteria can offer solid alternative solutions for the model selection problem in scenarios with limited observability andnoisy data.

However, domain specific criteria for model selection should always be carefully chosen, based on solid background, and their influence on the final selection decision should be carefully weighted. Combined with global heuristic parameter estimation approaches, as used in this study, inattentively chosen criteria might shift the solution to an unwanted direction. Incorrect weighting, on the other hand, might aggravate the selection problem by under– or over-fitting of the candidate models.

## Conclusion

We have demonstrated the applicability of the automated modeling tool ProBMoT to the real-world problem of modeling the Rab5-Rab7 conversion switch in the important cellular process of endocytosis. By using ProBMoT, we improve upon the classical modeling approach by using domain-specific knowledge, good practices, and automation. While the applicability of ProBMoT and other modeling approaches has been illustrated before [13], this is the first study focusing on the problem of model selection. In these terms, we go beyond the work of Čerepnalkoski et al. [17] and Tashkova et al. [16] and make a step further towards elucidating the problem of model selection in the context of automated modeling of dynamical systems.

Furthermore, we show that ProBMoT is able, in an automated fashion and using a combination of knowledge– and data-driven modeling, to solve a complex, relevant and challenging problem from the domain of systems biology. We analyze its utility by comparing the results of automated modeling with the ones obtained in a manual modeling experiment. In this way, we evaluate both the automated approach and the manual modeling process. The results show that ProBMoT is able to reconstruct the results of the manual experiment by using limited and noisy observations of the modeled system. The modeling experiments presented here confirm the finding that a group of model structures able to achieve a cut-out or toggle switch behavior explains the available data. We also show that another group of model structures (IP group), previously considered less plausible, and a number of previously not considered model structures, are still equally capable of reproducing the observations and expectations and should still be consideredas relevant.

We identify several points for further work. Additional criteria, more complex than the considered one, which complement the information about the model fit to the measured data should be considered. Such criteria can be based on the properties of the model structure: Del Conte-Zerial et al. [15] perform e.g. bifurcation and phase plane analysis on each model structure, after which they dismiss the structures that lack certainproperties.

A similar effect can be achieved by apriori filtering of candidate model structures based on their structural properties. The constraining of the domain knowledge based on valid assumptions and the introduction of specific knowledge related to the problem at hand, will result in a reduced number of candidate models to be fitted. This will reduce the computational time needed for the experiments and facilitate the model selection problem. However, as shown trough our experiments, with the use of a domain-specific criteria, the automated process-based modeling achieves high selectivity even in the presence of unfiltered model structures.

Finally, the automated modeling approach can be used to gain knowledge about other dynamical systems, i.e., other parts of the endocytic pathway. The gained knowledge can contribute to the development of a complete explanatory model of endocytosis. By performing experiments on other real-world problems, additional insight into the process of automated modeling can be obtained. This will further improve the used approaches, which can in turn be used to discover better explanatory models.

## Additional files

Additional file 1 **Supplementary material.** This file contains supplemental figures and tables.

Additional file 2 **ProBMoT Library, Incomplete Model and Task files.** This file contains the complete library, the incomplete model and the task used for modeling the Rab5-Rab7 switch.

Additional file 3 **Data.** This file contains space delimited time points and measurements used for fitting the model structures.

## Abbreviations

ProBMoT: Process Based Modeling Tool
GDP: Guanosine diphosphate
GTP: Guanosine triphosphate
GDI: Guanosise diphosphate Dissociation Inhibitor
GEF: Guanine nucleotide Exchange Factor
GAP: GTPase-Activating Protein
COT: Cut-Out/Toggle switch
IP: In-Phase switch
NOBS: NO Bistable Switch
