# Supplementary material for: Domain-specific model selection for structural identification of the Rab5-Rab7 dynamics in endocytosis
Source: BMC Syst Biol. 2015 Jun 26;9:31. doi: 10.1186/s12918-015-0175-x (PMC4482292; doi:10.1186/s12918-015-0175-x)
Supplement: Supplementary file 1 — Supplementary material. This file contains supplemental figures and tables. [file 12918_2015_175_MOESM1_ESM.pdf]

Domain-specific model selection for structural identification of the Rab5-Rab7 dynamics in endocytosis –Supplementary material–

Jovan Tanevski, Ljupčo Todorovski, Yannis Kalaidzidis, Sašo Džeroski

Supplemental figures

Figure 1 – Distribution of the structural components of all 126 candidate models considered in the experiments, as defined in the library. 4
Figure 2 – Plot of the sorted complexities of the 126 candidate models. $C(m)$ represents the normalized complexity of the model. The complexity in the histogram is expressed in terms of the number of interactions present in the model. 5
Figure 3 – The error profile obtained using the ER criterion and a trade-off parameter setting $\alpha = 0.5$ (left). The box plot shows the distribution of error in the profile (right). 6
Figure 4 – The error profile obtained using the EX criterion and a trade-off parameter setting $\alpha = 0.9$ (left). The box plot shows the distribution of error in the profile (right). 7
Figure 5 – The error profile obtained using the ERX criterion and a trade-off parameter setting $\alpha = 0.5$ (left). The box plot shows the distribution of error in the profile (right). 8
Figure 6 – Box plots of the distribution of errors in terms of E, ER (top) and EX, ERX (bottom) relative to the complexity C of the model structures. The correlations between the errors and the complexity of the model structures are as follows: $\rho(E, C) = -0.110$, $\rho(ER, C) = -0.159$, $\rho(EX, C) = 0.184$, $\rho(ERX, C) = 0.168$, where ρ is the Spearman's rank correlation coefficient. 11
Figure 7 – Correlation matrix for the parameters of the overall top-ranked model obtained using the bootstrap method. The names of the parameters for each row/column are shown in the main diagonal. The matrix is symmetric. Above the main diagonal, the values of the correlations correspond to the Pearson correlation coefficient. . 13
Figure 8 – Histograms of the estimated parameter values of the overall top-ranked model obtained using the bootstrap method. In each histogram, the yellow horizontal line represents the 95% confidence interval and the green vertical line represents the mean of the outliers-filtered sample. The width of the bins is calculated according to the Freedman-Diaconis rule. 14

Figure 9 – Correlation matrix for the parameters of the top-ranked model from the COT group obtained using the bootstrap method. The names of the parameters for each row/column are shown in the main diagonal. The matrix is symmetric. Above the main diagonal, the values of the correlations correspond to the Pearson correlation coefficient. 16
Figure 10 – Histograms of the estimated parameter values of the top-ranked model from the COT group obtained using the bootstrap method. In each histogram, the yellow horizontal line represents the 95% confidence interval and the green vertical line represents the mean of the outliers-filtered sample. The width of the bins is calculated according to the Freedman-Diaconis rule. 17
Figure 11 – Correlation matrix for the parameters of the top-ranked model from the IP group obtained using the bootstrap method. The names of the parameters for each row/column are shown in the main diagonal. The matrix is symmetric. Above the main diagonal, the values of the correlations correspond to the Pearson correlation coefficient. 19
Figure 12 – Histograms of the estimated parameter values of the top-ranked model from the IP group obtained using the bootstrap method. In each histogram, the yellow horizontal line represents the 95% confidence interval and the green vertical line represents the mean of the outliers-filtered sample. The width of the bins is calculated according to the Freedman-Diaconis rule. 20

Supplemental tables

Table 1 – Detailed description of the structure and the performance of the models in the plateau obtained using the ERX criterion with $\alpha = 0.5$. 9
Table 2 – Detailed description of the structure and the performance of the most complex and least complex models in the plateau obtained using the ERX criterion with $\alpha = 0.5$. 10
Table 3 – Summary of the identifiability analysis for the estimated parameter values in the overall top-ranked model using the bootstrap method. The column estimate contains the values in the model obtained using ProBMoT. The column Mean contains the mean of the outliers-filtered sample. The C^{lo} and C^{hi} columns contain the lower and upper bound of the 95% confidence interval. The C^{L} and C^{sh} columns contain the length and the shape of the confidence interval, where $C^{L} = C^{lo} - C^{hi}$ and $C^{sh} = \frac{C^{hi} - Mean}{Mean - C^{lo}}$. Assuming a confidence interval for a normal distribution, $C^{sh} = 1$ and the interval is symmetric about the mean, the column outliers contains the number of outliers from a sample of 1000. 12
Table 4 – Summary of the identifiability analysis for the estimated parameter values in the top-ranked model from the COT group using the bootstrap method. The column estimate contains the values in the model obtained using ProBMoT. The column Mean contains the mean of the outliers-filtered sample. The C^{lo} and C^{hi} columns contain the lower and upper bound of the 95% confidence interval. The C^{L} and C^{sh} columns contain the length and the shape of the confidence interval, where $C^{L} = C^{lo} - C^{hi}$ and $C^{sh} = \frac{C^{hi} - Mean}{Mean - C^{lo}}$. Assuming a confidence interval for a normal distribution, $C^{sh} = 1$ and the interval is symmetric about the mean, the column outliers contains the number of outliers from a sample of 1000. 15

Table 5 – Summary of the identifiability analysis for the estimated parameter values in the top-ranked model from the IP group using the bootstrap method. The column estimate contains the values in the model obtained using ProBMoT. The column Mean contains the mean of the outliers-filtered sample. The C^{lo} and C^{hi} columns contain the lower and upper bound of the 95% confidence interval. The C^{L} and C^{sh} columns contain the length and the shape of the confidence interval, where $C^{L} = C^{lo} - C^{hi}$ and $C^{sh} = \frac{C^{hi} - Mean}{Mean - C^{lo}}$. Assuming a confidence interval for a normal distribution, $C^{sh} = 1$ and the interval is symmetric about the mean, the column outliers contains the number of outliers from a sample of 1000. 18

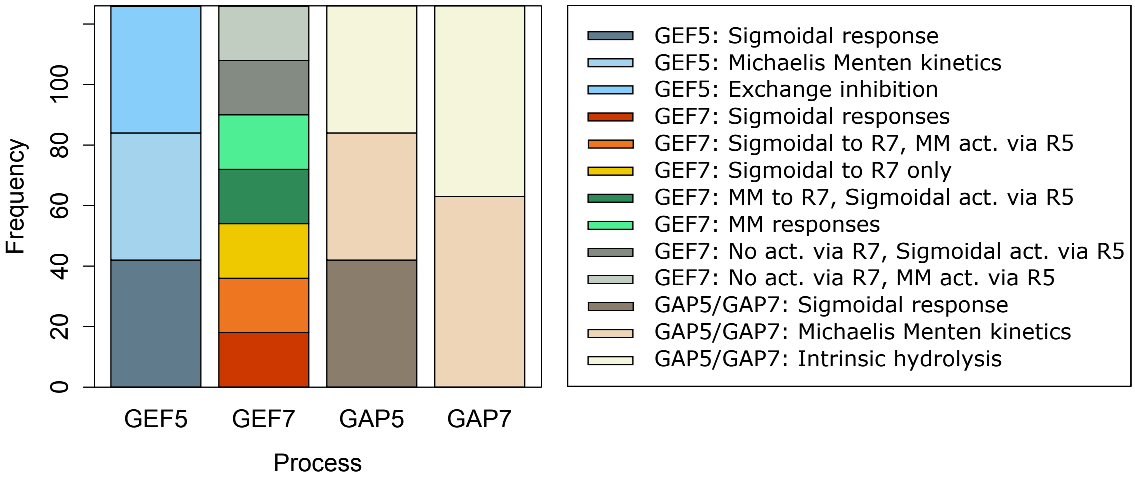

Figure 1: Distribution of the structural components of all 126 candidate models considered in the experiments, as defined in the library.

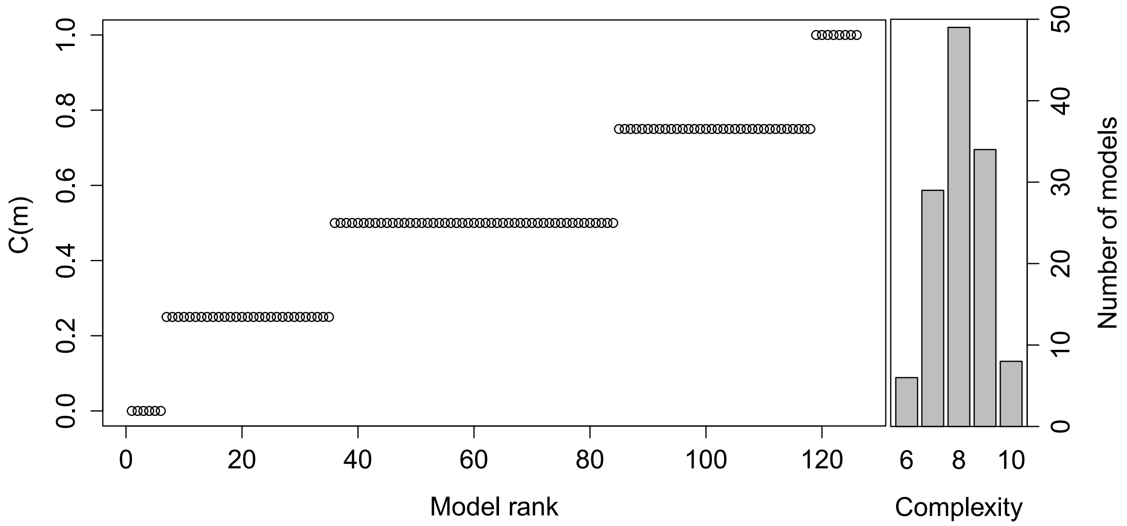

Figure 2: Plot of the sorted complexities of the 126 candidate models. $C(m)$ represents the normalized complexity of the model. The complexity in the histogram is expressed in terms of the number of interactions present in the model.

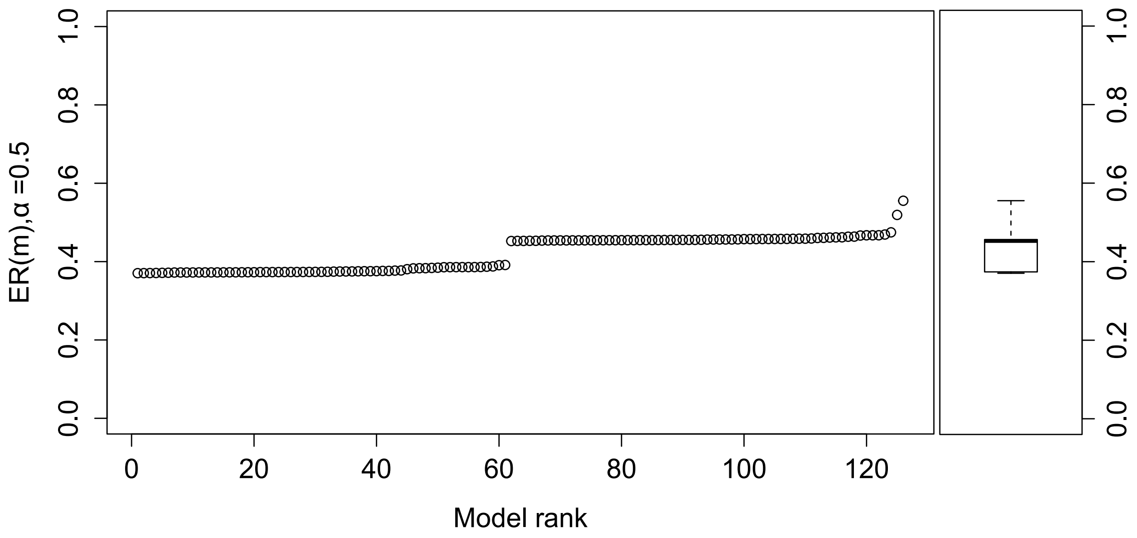

Figure 3: The error profile obtained using the ER criterion and a trade-off parameter setting $\alpha = 0.5$ (left). The box plot shows the distribution of error in the profile (right).

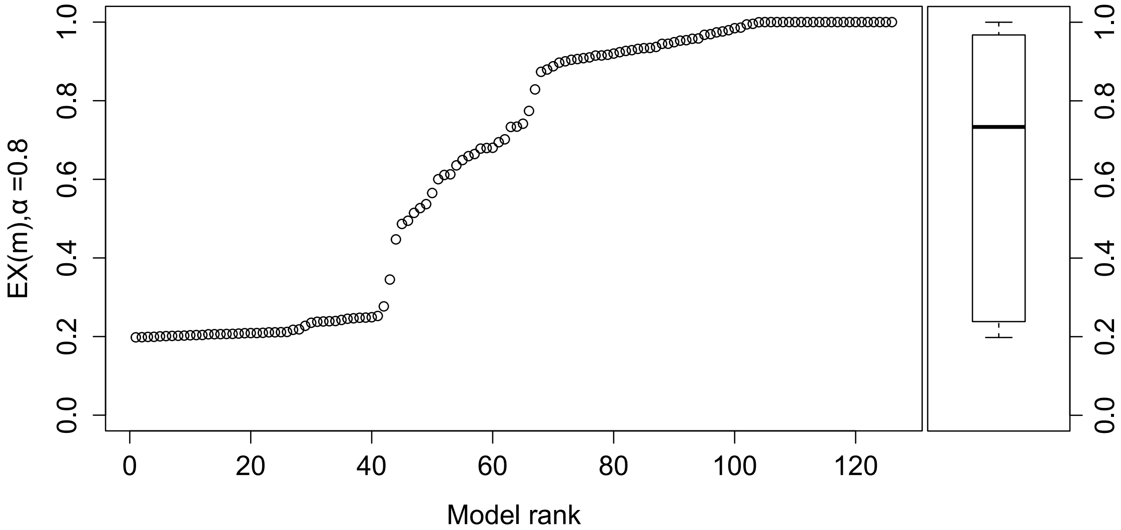

Figure 4: The error profile obtained using the EX criterion and a trade-off parameter setting $\alpha = 0.9$ (left). The box plot shows the distribution of error in the profile (right).

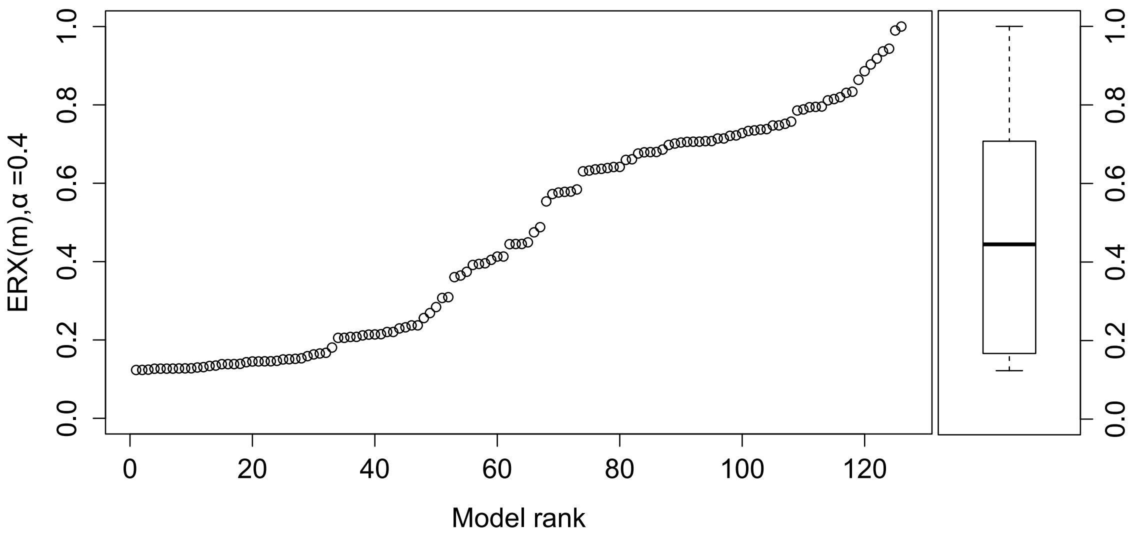

Figure 5: The error profile obtained using the ERX criterion and a trade-off parameter setting $\alpha = 0.5$ (left). The box plot shows the distribution of error in the profile (right).

Table 1: Detailed description of the structure and the performance of the models in the plateau obtained using the ERX criterion with $\alpha = 0.5$.

Rank	Model	Group	Structure					Performance				C
			GEF5	GAP5	GEF7 auto catalysis	GEF7 via Rab5	GAP7	E	R	X	ERX	
**1**	**74**	**OTHER**	**Sigmoidal_response**	**Intrinsic_hydrolysis**	**Sigmoidal_response**	**None**	**MMenten**	**0.224**	**0.045**	**0.010**	**0.126**	**0.50**
2	77	OTHER	Sigmoidal_response	Intrinsic_hydrolysis	Sigmoidal_response	None	MMenten	0.224	0.045	0.010	0.126	0.25
3	104	OTHER	Sigmoidal_response	MMenten	None	Sigmoidal_response	MMenten	0.226	0.045	0.010	0.127	0.50
**4**	**86**	**COT**	**Sigmoidal_response**	**MMenten**	**MMenten_kinetics**	**MMenten_kinetics**	**MMenten**	**0.233**	**0.040**	**0.010**	**0.129**	**0.75**
**5**	**23**	**IP**	**Sigmoidal_response**	**MMenten**	**MMenten_kinetics**	**MMenten_kinetics**	**Intrinsic_hydrolysis**	**0.230**	**0.047**	**0.010**	**0.129**	**0.50**
6	101	OTHER	Sigmoidal_response	MMenten	None	MMenten_kinetics	MMenten	0.228	0.050	0.010	0.129	0.50
7	41	IP	Sigmoidal_response	MMenten	None	Sigmoidal_response	Intrinsic_hydrolysis	0.231	0.045	0.010	0.129	0.25
8	71	OTHER	Sigmoidal_response	Intrinsic_hydrolysis	Sigmoidal_response	MMenten_kinetics	MMenten	0.232	0.041	0.010	0.129	0.50
9	38	IP	Sigmoidal_response	MMenten	None	MMenten_kinetics	Intrinsic_hydrolysis	0.233	0.044	0.010	0.130	0.25
10	60	OTHER	Exchange_inhibition	Sigmoidal	None	MMenten_kinetics	Intrinsic_hydrolysis	0.233	0.044	0.010	0.130	0.50
11	59	IP	Sigmoidal_response	Sigmoidal	None	MMenten_kinetics	Intrinsic_hydrolysis	0.234	0.051	0.010	0.132	0.25
12	125	OTHER	Sigmoidal_response	Sigmoidal	None	Sigmoidal_response	MMenten	0.234	0.051	0.010	0.132	0.50
13	29	COT	Sigmoidal_response	Sigmoidal	Sigmoidal_response	MMenten_kinetics	Intrinsic_hydrolysis	0.241	0.046	0.010	0.134	0.50
14	8	COT	Sigmoidal_response	Intrinsic_hydrolysis	Sigmoidal_response	MMenten_kinetics	Intrinsic_hydrolysis	0.247	0.042	0.010	0.137	0.25
15	44	IP	Sigmoidal_response	Sigmoidal	MMenten_kinetics	MMenten_kinetics	Intrinsic_hydrolysis	0.254	0.044	0.010	0.140	0.50
16	83	OTHER	Sigmoidal_response	Intrinsic_hydrolysis	None	Sigmoidal_response	MMenten	0.259	0.035	0.010	0.141	0.25
17	98	OTHER	Sigmoidal_response	MMenten	Sigmoidal_response	None	MMenten	0.259	0.035	0.010	0.141	0.50
18	95	OTHER	Sigmoidal_response	MMenten	Sigmoidal_response	Sigmoidal_response	MMenten	0.260	0.036	0.010	0.142	0.75
19	107	COT	Sigmoidal_response	Sigmoidal	MMenten_kinetics	MMenten_kinetics	MMenten	0.266	0.041	0.010	0.145	0.75
20	119	OTHER	Sigmoidal_response	Sigmoidal	Sigmoidal_response	None	MMenten	0.260	0.041	0.037	0.150	0.50
21	26	IP	Sigmoidal_response	MMenten	MMenten_kinetics	Sigmoidal_response	Intrinsic_hydrolysis	0.258	0.064	0.010	0.148	0.50
22	5	IP	Sigmoidal_response	Intrinsic_hydrolysis	MMenten_kinetics	Sigmoidal_response	Intrinsic_hydrolysis	0.259	0.063	0.010	0.148	0.25
23	122	OTHER	Sigmoidal_response	Sigmoidal	None	Sigmoidal_response	MMenten	0.270	0.039	0.010	0.148	0.50
24	116	OTHER	Sigmoidal_response	Sigmoidal	Sigmoidal_response	Sigmoidal_response	MMenten	0.273	0.04	0.010	0.149	0.75
25	50	COT	Sigmoidal_response	Sigmoidal	Sigmoidal_response	MMenten_kinetics	Intrinsic_hydrolysis	0.277	0.045	0.010	0.152	0.50
26	56	COT	Sigmoidal_response	Sigmoidal	Sigmoidal_response	None	Intrinsic_hydrolysis	0.281	0.041	0.010	0.153	0.25
27	113	OTHER	Sigmoidal_response	Sigmoidal	Sigmoidal_response	MMenten_kinetics	MMenten	0.282	0.040	0.010	0.154	0.75
28	110	COT	Sigmoidal_response	Sigmoidal	MMenten_kinetics	Sigmoidal_response	MMenten	0.282	0.041	0.010	0.154	0.75
29	53	COT	Sigmoidal_response	Sigmoidal	Sigmoidal_response	Sigmoidal_response	Intrinsic_hydrolysis	0.240	0.154	0.010	0.161	0.50
30	14	COT	Sigmoidal_response	Intrinsic_hydrolysis	Sigmoidal_response	None	Intrinsic_hydrolysis	0.243	0.166	0.010	0.166	0.00
31	4	OTHER	Mmenten_kinetics	Intrinsic_hydrolysis	MMenten_kinetics	Sigmoidal_response	Intrinsic_hydrolysis	0.306	0.050	0.010	0.168	0.25
32	35	COT	Sigmoidal_response	MMenten	Sigmoidal_response	None	Intrinsic_hydrolysis	0.247	0.172	0.010	0.169	0.25
33	124	OTHER	MMenten_kinetics	Sigmoidal	None	Sigmoidal_response	MMenten	0.325	0.062	0.037	0.187	0.50

Table 2: Detailed description of the structure and the performance of the most complex and least complex models in the plateau obtained using the ERX criterion with $\alpha = 0.5$.

Rank	Model	Group	Structure					Performance				C
			GEF5	GAP5	GEF7 auto catalysis	GEF7 via Rab5	GAP7	E	R	X	ERX	
30	14	COT	Sigmoidal_response	Intrinsic_hydrolysis	Sigmoidal_response	None	Intrinsic_hydrolysis	0.242	0.166	0.010	0.166	0.00
38	17	IP	Sigmoidal_response	Intrinsic_hydrolysis	None	MMenten_kinetics	Intrinsic_hydrolysis	0.401	0.044	0.010	0.214	0.00
43	20	IP	Sigmoidal_response	Intrinsic_hydrolysis	None	Sigmoidal_response	Intrinsic_hydrolysis	0.406	0.069	0.010	0.223	0.00
69	111	OTHER	Exchange_Inhibition	Sigmoidal	MMenten_kinetics	Sigmoidal_response	MMenten	1.000	0.200	0.080	0.573	1.00
82	114	OTHER	Exchange_Inhibition	Sigmoidal	Sigmoidal_response	MMenten_kinetics	MMenten	1.163	0.240	0.080	0.662	1.00
86	93	OTHER	Exchange_Inhibition	MMenten	Sigmoidal_response	MMenten_kinetics	MMenten	1.188	0.272	0.075	0.681	1.00
92	96	OTHER	Exchange_Inhibition	MMenten	Sigmoidal_response	Sigmoidal_response	MMenten	1.054	0.636	0.081	0.710	1.00
95	16	OTHER	MMenten_kinetics	Intrinsic_hydrolysis	None	MMenten_kinetics	Intrinsic_hydrolysis	0.982	0.787	0.080	0.710	0.00
96	117	OTHER	Exchange_Inhibition	Sigmoidal	Sigmoidal_response	Sigmoidal_response	MMenten	0.999	0.780	0.080	0.714	1.00
99	19	OTHER	MMenten_kinetics	Intrinsic_hydrolysis	None	Sigmoidal_response	Intrinsic_hydrolysis	1.183	0.440	0.081	0.722	0.00
114	87	OTHER	Exchange_Inhibition	MMenten	MMenten_kinetics	MMenten_kinetics	MMenten	1.197	0.772	0.080	0.812	1.00
116	13	OTHER	MMenten_kinetics	Intrinsic_hydrolysis	Sigmoidal_response	None	Intrinsic_hydrolysis	1.152	0.900	0.076	0.820	0.00
124	108	OTHER	Exchange_Inhibition	Sigmoidal	MMenten_kinetics	MMenten_kinetics	MMenten	1.130	1.000	1.000	1.070	1.00
125	90	OTHER	Exchange_Inhibition	MMenten	MMenten_kinetics	Sigmoidal_response	MMenten	1.172	1.000	1.000	1.086	1.00

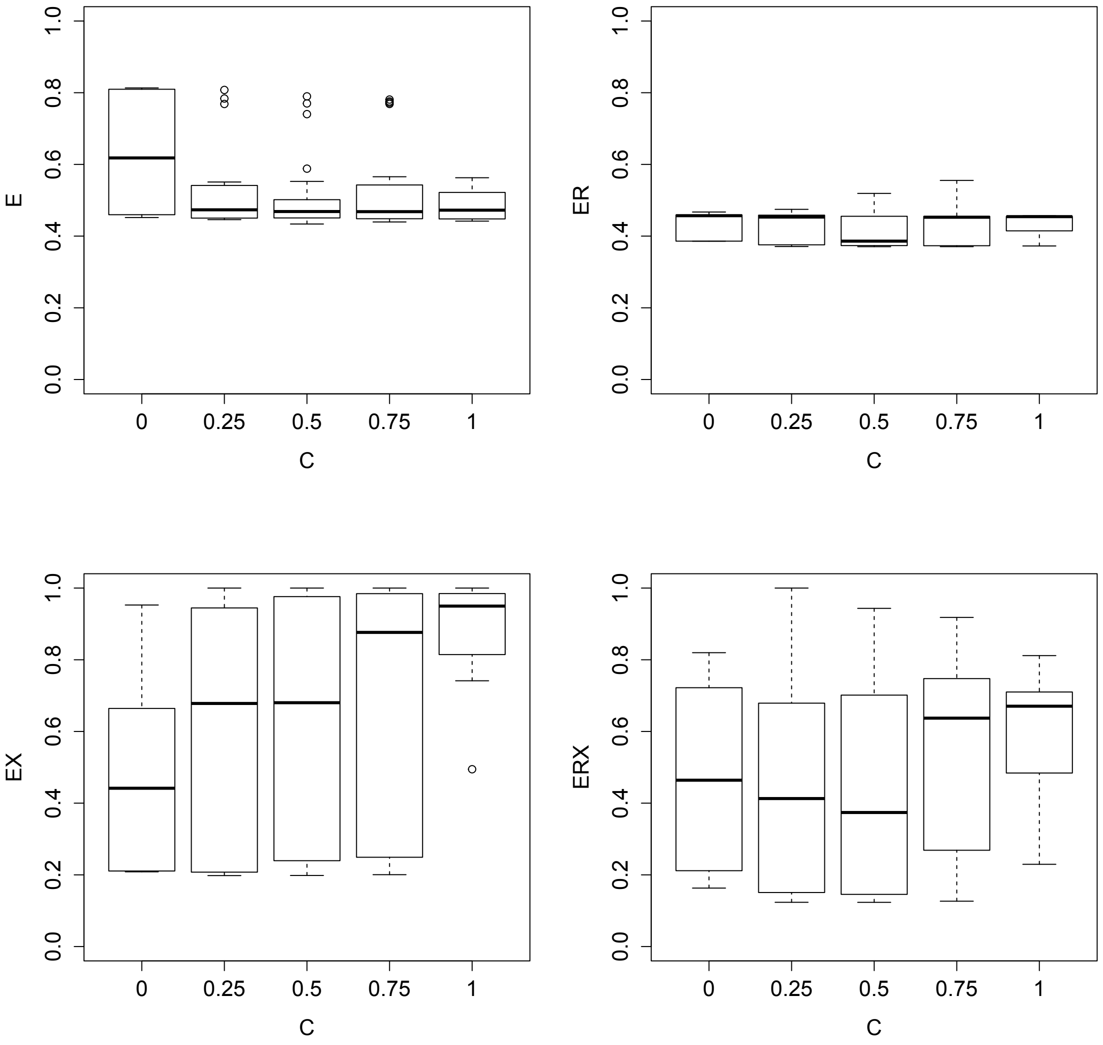

Figure 6: Box plots of the distribution of errors in terms of E, ER (top) and EX, ERX (bottom) relative to the complexity C of the model structures. The correlations between the errors and the complexity of the model structures are as follows: $\rho(E, C) = -0.110$, $\rho(ER, C) = -0.159$, $\rho(EX, C) = 0.184$, $\rho(ERX, C) = 0.168$, where ρ is the Spearman's rank correlation coefficient.

Top ranked model:

$$
\begin{aligned}
\frac{dr_5}{dt} &= K_1 - (k_1 + \frac{k_{e,5}}{1+e^{(k_{g,5}-R_5)\cdot k_{f,5}}}) \cdot r_5 + k_{h,5} \cdot R_5 \\
\frac{dR_5}{dt} &= \frac{k_{e,5}}{1+e^{(k_{g,5}-R_5)\cdot k_{f,5}}} \cdot r_5 - k_{h,5} \cdot R_5 \\
\frac{dr_7}{dt} &= K_2 - (k_2 + \frac{k_{e,7}}{1+e^{(k_{g,7}-R_7)\cdot k_{f,7}}} + \frac{k_{E,7}}{1+e^{(k_{G,7}-R_5)\cdot k_{F,7}}}) \cdot r_7 + k_{h,7} + \frac{k_{H,7} \cdot R_5}{k_{y,7}+R_5} \cdot R_7 \\
\frac{dR_7}{dt} &= \frac{k_{e,7}}{1+e^{(k_{g,7}-R_7)\cdot k_{f,7}}} + \frac{k_{E,7}}{1+e^{(k_{G,7}-R_5)\cdot k_{F,7}}} \cdot r_7 - k_{h,7} + \frac{k_{H,7} \cdot R_5}{k_{y,7}+R_5} \cdot R_7
\end{aligned}
\tag{1}
$$

Table 3: Summary of the identifiability analysis for the estimated parameter values in the overall top-ranked model using the bootstrap method. The column estimate contains the values in the model obtained using ProBMoT. The column Mean contains the mean of the outliers-filtered sample. The C^{lo} and C^{hi} columns contain the lower and upper bound of the 95% confidence interval. The C^L and C^{sh} columns contain the length and the shape of the confidence interval, where $C^L = C^{lo} - C^{hi}$ and $C^{sh} = \frac{C^{hi}-Mean}{Mean-C^{lo}}$. Assuming a confidence interval for a normal distribution, $C^{sh} = 1$ and the interval is symmetric about the mean, the column outliers contains the number of outliers from a sample of 1000.

Parameter	Estimate	Mean	C^{lo}	C^{hi}	C^L	C^{sh}	Outliers
r5(0)	2	1.892	1.555	2	0.445	0.318	143
R5(0)	1.603	1.511	1.308	1.747	0.438	1.164	191
K1	0.098	0.132	0.111	0.155	0.044	1.132	209
k1	0.119	0.139	0.122	0.158	0.036	1.094	163
r7(0)	0.097	0.187	0	0.485	0.485	1.590	27
R7(0)	0.305	0.107	0	0.310	0.310	1.888	5
K2	0.141	0.223	0.001	0.623	0.622	1.800	141
k2	0.093	1.132	0.001	3.772	3.771	2.333	0
td	31.744	104.918	5	195	190	0.901	0
ke,5	0.001	0.001	0.001	0.001	0.000	11.104	192
kf,5	0.744	2.750	0.001	4	3.999	0.454	0
kg,5	3.317	3.320	0.914	4	3.085	0.282	79
ke,7	2.488	1.888	0.001	4	3.999	1.118	0
kf,7	0.001	1.174	0.001	4	3.999	2.406	0
kg,7	0.675	1.868	0.001	4	3.999	1.141	0
kh,5	0.013	0.013	0.013	0.014	0.001	1.193	170
kH,7	3.144	0.710	0.001	2.071	2.070	1.919	19
ky,7	4	3.714	2.830	4	1.169	0.323	99
kh,7	2.946	0.497	0.001	1.218	1.217	1.450	3
kE,7	3.890	3.271	1.754	4	2.245	0.479	82
kF,7	3.821	2.007	0.001	4	3.999	0.992	0
kG,7	0.142	0.742	0.001	2.988	2.987	3.031	94
K	7946.916	8304.990	7287.316	9371.584	2084.267	1.048	107

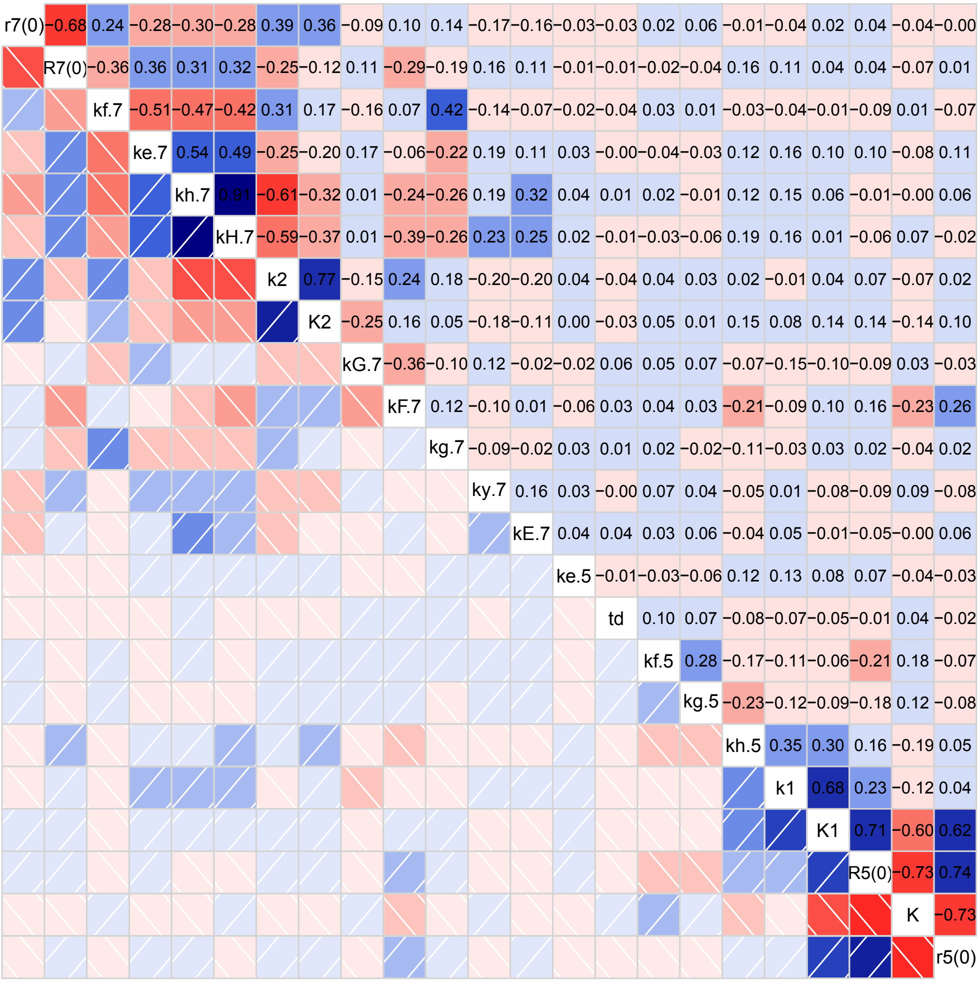

Figure 7: Correlation matrix for the parameters of the overall top-ranked model obtained using the bootstrap method. The names of the parameters for each row/column are shown in the main diagonal. The matrix is symmetric. Above the main diagonal, the values of the correlations correspond to the Pearson correlation coefficient.

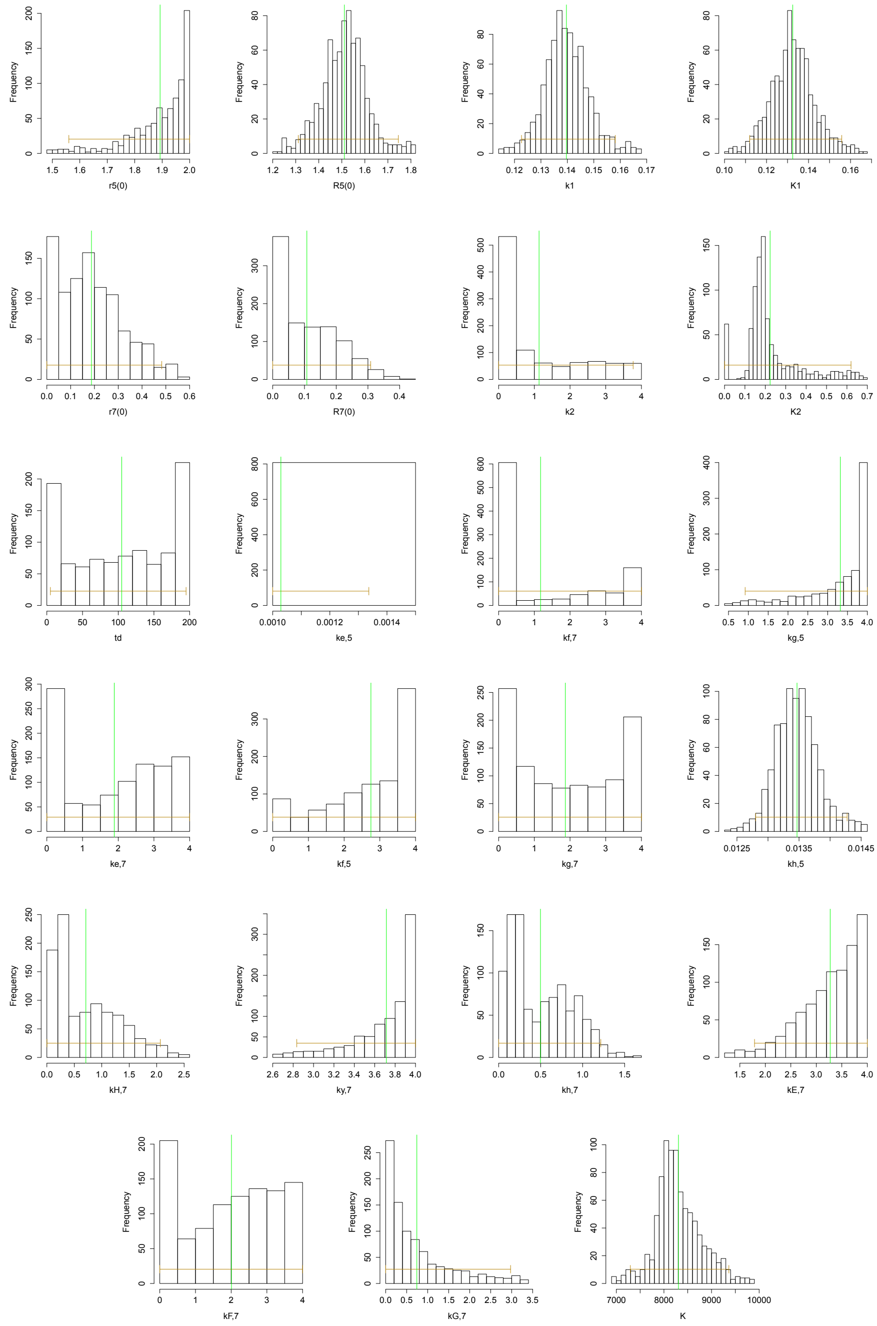

Figure 8: Histograms of the estimated parameter values of the overall top-ranked model obtained using the bootstrap method. In each histogram, the yellow horizontal line represents the 95% confidence interval and the green vertical line represents the mean of the outliers-filtered sample. The width of the bins is calculated according to the Freedman-Diaconis rule.

Top ranked COT model:

$$\begin{aligned}
\frac{dr_5}{dt} &= K_1 - (k_1 + \frac{k_{e,5}}{1+e^{(k_{g,5}-R_5)\cdot k_{f,5}}}) \cdot r_5 + k_{h,5} + \frac{k_{H,5}\cdot R_7}{k_{y,5}+R_7} \cdot R_5 \\
\frac{dR_5}{dt} &= \frac{k_{e,5}}{1+e^{(k_{g,5}-R_5)\cdot k_{f,5}}} \cdot r_5 - k_{h,5} + \frac{k_{H,5}\cdot R_7}{k_{y,5}+R_7} \cdot R_5 \\
\frac{dr_7}{dt} &= K_2 - (k_2 + \frac{k_{e,7}\cdot R_7}{k_{g,5}+R_7} + \frac{k_{E,7}\cdot R_5}{k_{G,7}+R_5}) \cdot r_7 + k_{h,7} + \frac{k_{H,7}\cdot R_5}{k_{y,7}+R_5} \cdot R_7 \\
\frac{dR_7}{dt} &= \frac{k_{e,7}\cdot R_7}{k_{g,5}+R_7} + \frac{k_{E,7}\cdot R_5}{k_{G,7}+R_5} \cdot r_7 - k_{h,7} + \frac{k_{H,7}\cdot R_5}{k_{y,7}+R_5} \cdot R_7
\end{aligned} \tag{2}$$

Table 4: Summary of the identifiability analysis for the estimated parameter values in the top-ranked model from the COT group using the bootstrap method. The column estimate contains the values in the model obtained using ProBMoT. The column Mean contains the mean of the outliers-filtered sample. The C^{lo} and C^{hi} columns contain the lower and upper bound of the 95% confidence interval. The C^L and C^{sh} columns contain the length and the shape of the confidence interval, where $C^L = C^{lo} - C^{hi}$ and $C^{sh} = \frac{C^{hi}-Mean}{Mean-C^{lo}}$. Assuming a confidence interval for a normal distribution, $C^{sh} = 1$ and the interval is symmetric about the mean, the column outliers contains the number of outliers from a sample of 1000.

Parameter	Estimate	Mean	C^{lo}	C^{hi}	C^L	C^{sh}	Outliers
r5(0)	1.201	1.491	0.085	2	1.914	0.361	10
R5(0)	1.173	1.217	0	2	2	0.643	0
K1	0.073	0.097	0.001	0.177	0.176	0.842	102
k1	0.156	0.138	0.099	0.180	0.081	1.088	322
r7(0)	0.100	0.236	0	0.800	0.800	2.391	72
R7(0)	0.132	0.089	0	0.345	0.345	2.884	51
K2	0.160	0.238	0.001	0.721	0.720	2.040	75
k2	0.222	1.199	0.001	3.985	3.984	2.324	0
td	20.169	87.954	5	195	190	1.290	0
ke,5	0.010	0.002	0.001	0.009	0.008	9.104	244
kf,5	0.714	2.481	0.001	4	3.999	0.612	0
kg,5	0.351	2.650	0.001	4	3.999	0.509	0
ke,7	0.096	1.233	0.001	4	3.999	2.245	0
kg,7	3.659	1.192	0.001	4	3.999	2.355	0
kH,5	0.002	0.005	0.001	0.015	0.014	2.128	180
ky,5	0.413	0.959	0.001	4	3.999	3.171	0
kH,7	1.695	0.579	0.001	2.212	2.211	2.825	78
ky,7	3.754	2.576	0.001	4	3.999	0.552	0
kh,7	0.739	0.215	0.001	0.840	0.839	2.916	36
kE,7	3.822	1.508	0.001	3.983	3.982	1.642	0
kG,7	1.229	2.021	0.001	4	3.999	0.979	0
kh,5	0.013	0.008	0.001	0.016	0.015	1.097	218
K	12330.653	9005.032	7121.954	15383.476	8261.522	3.387	200

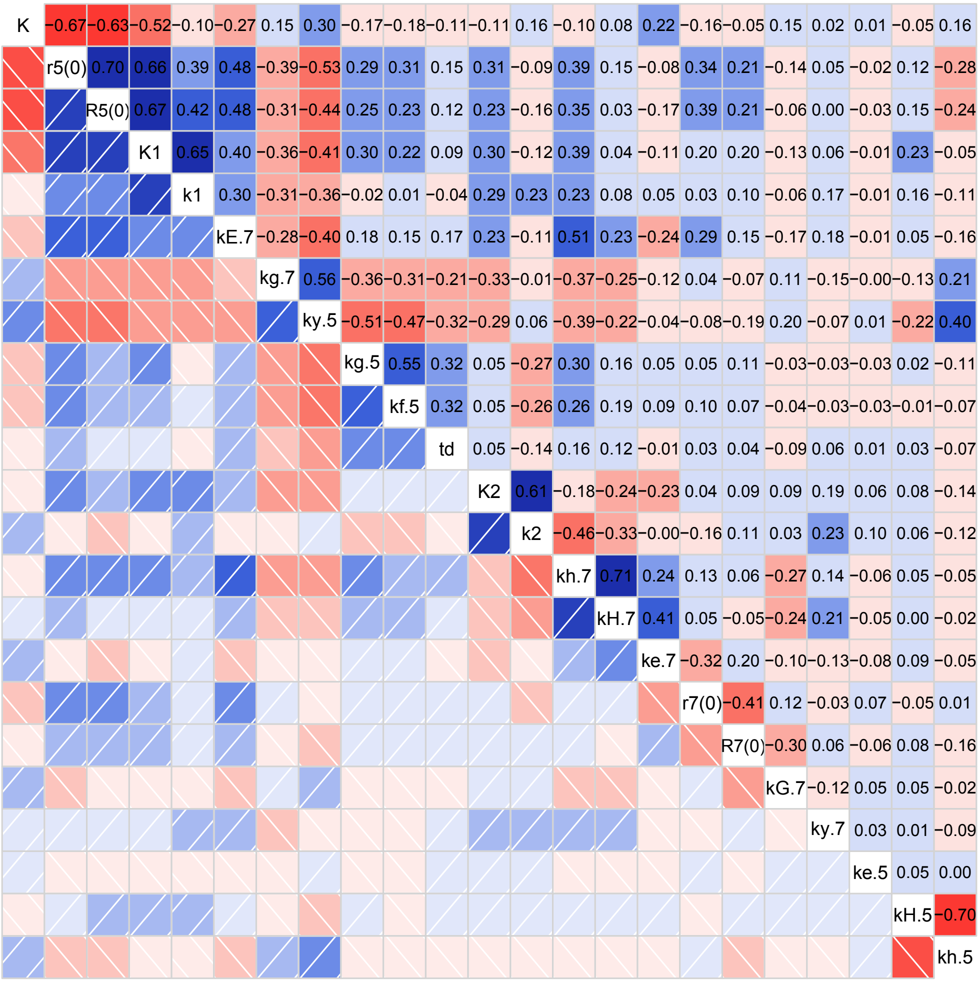

Figure 9: Correlation matrix for the parameters of the top-ranked model from the COT group obtained using the bootstrap method. The names of the parameters for each row/column are shown in the main diagonal. The matrix is symmetric. Above the main diagonal, the values of the correlations correspond to the Pearson correlation coefficient.

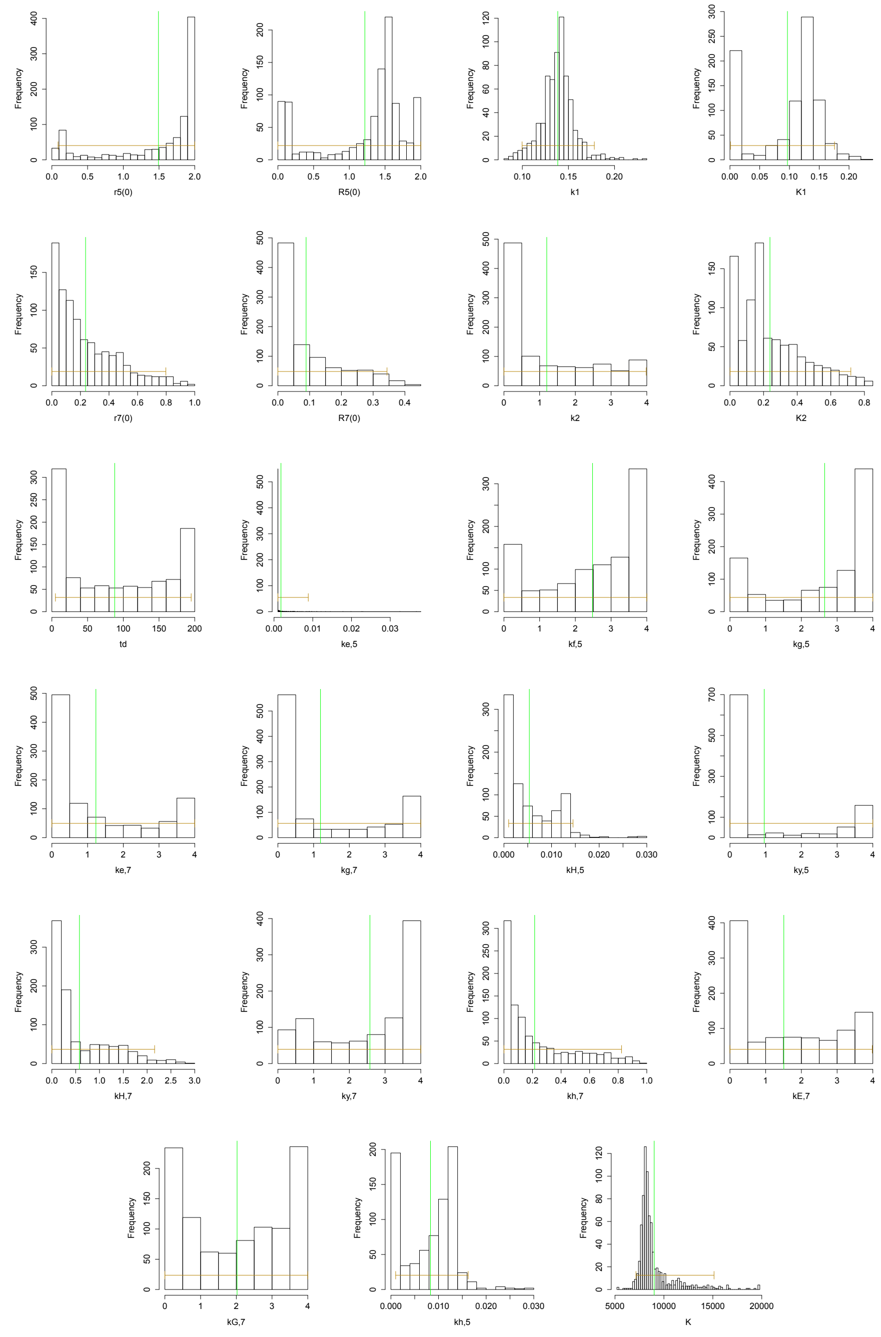

Figure 10: Histograms of the estimated parameter values of the top-ranked model from the COT group obtained using the bootstrap method. In each histogram, the yellow horizontal line represents the 95% confidence interval and the green vertical line represents the mean of the outliers-filtered sample. The width of the bins is calculated according to the Freedman-Diaconis rule.

Top ranked IP model:

$$
\begin{aligned}
\frac{dr_5}{dt} &= K_1 - (k_1 + \frac{k_{e,5}}{1 + e^{(k_{g,5} - R_5) \cdot k_{f,5}}}) \cdot r_5 + k_{h,5} + \frac{k_{H,5} \cdot R_7}{k_{y,5} + R_7} \cdot R_5 \\
\frac{dR_5}{dt} &= \frac{k_{e,5}}{1 + e^{(k_{g,5} - R_5) \cdot k_{f,5}}} \cdot r_5 - k_{h,5} + \frac{k_{H,5} \cdot R_7}{k_{y,5} + R_7} \cdot R_5 \\
\frac{dr_7}{dt} &= K_2 - (k_2 + \frac{k_{e,7} \cdot R_7}{k_{g,5} + R_7} + \frac{k_{E,7} \cdot R_5}{k_{G,7} + R_5}) \cdot r_7 + k_{h,7} \cdot R_7 \\
\frac{dR_7}{dt} &= \frac{k_{e,7} \cdot R_7}{k_{g,5} + R_7} + \frac{k_{E,7} \cdot R_5}{k_{G,7} + R_5} \cdot r_7 - k_{h,7} \cdot R_7
\end{aligned}
\tag{3}
$$

Table 5: Summary of the identifiability analysis for the estimated parameter values in the top-ranked model from the IP group using the bootstrap method. The column estimate contains the values in the model obtained using ProBMoT. The column Mean contains the mean of the outliers-filtered sample. The C^{lo} and C^{hi} columns contain the lower and upper bound of the 95% confidence interval. The C^L and C^{sh} columns contain the length and the shape of the confidence interval, where $C^L = C^{lo} - C^{hi}$ and $C^{sh} = \frac{C^{hi} - Mean}{Mean - C^{lo}}$. Assuming a confidence interval for a normal distribution, $C^{sh} = 1$ and the interval is symmetric about the mean, the column outliers contains the number of outliers from a sample of 1000.

Parameter	Estimate	Mean	C^{lo}	C^{hi}	C^L	C^{sh}	Outliers
r5(0)	2	0.875	0	2	2	1.283	0
R5(0)	1.553	0.927	0	2	2	1.156	0
K1	0.092	0.088	0.001	0.345	0.344	2.950	192
k1	0.117	1.718	0.013	4	3.987	1.337	0
r7(0)	0.002	0.110	0	0.504	0.504	3.551	113
R7(0)	0.580	0.123	0	0.537	0.537	3.341	48
K2	0.114	0.349	0.001	1.520	1.519	3.356	53
k2	0.075	1.565	0.001	3.999	3.998	1.556	0
td	5	74.272	5	195	190	1.742	0
ke,5	0.001	1.019	0.001	4	3.999	2.924	0
kf,5	1.356	1.871	0.001	4	3.999	1.138	0
kg,5	3.399	2.125	0.001	4	3.999	0.882	0
ke,7	0.754	0.579	0.001	2.322	2.321	3.010	69
kg,7	0.001	2.937	0.001	4	3.999	0.362	0
kH,5	0.002	0.005	0.001	0.019	0.018	3.509	221
ky,5	4	1.501	0.001	4	3.999	1.664	0
kh,7	1.259	0.008	0.001	0.046	0.045	5.798	166
kE,7	3.422	0.025	0.001	0.278	0.277	10.573	222
kG,7	3.915	2.190	0.001	4	3.999	0.826	0
kh,5	0.009	0.015	0.001	0.048	0.047	2.478	186
K	8118.054	22189.124	7476.843	81297.686	73820.843	4.017	124

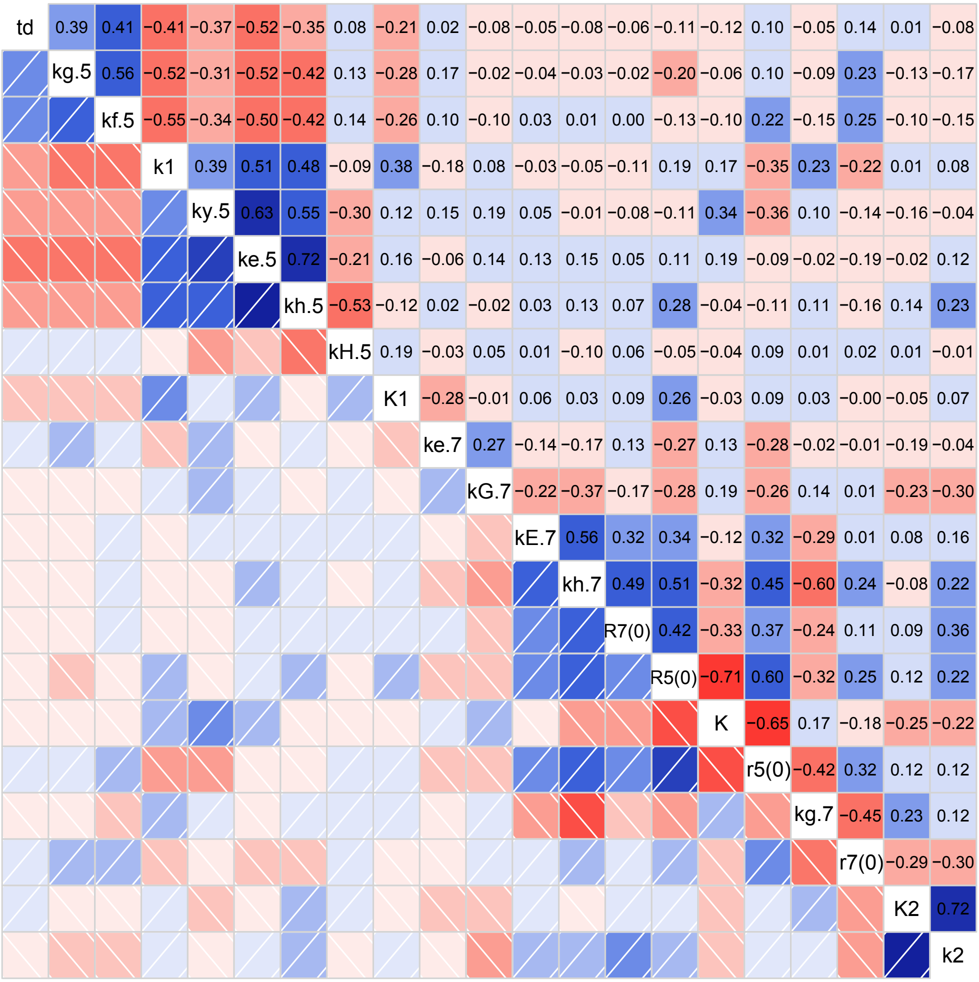

Figure 11: Correlation matrix for the parameters of the top-ranked model from the IP group obtained using the bootstrap method. The names of the parameters for each row/column are shown in the main diagonal. The matrix is symmetric. Above the main diagonal, the values of the correlations correspond to the Pearson correlation coefficient.

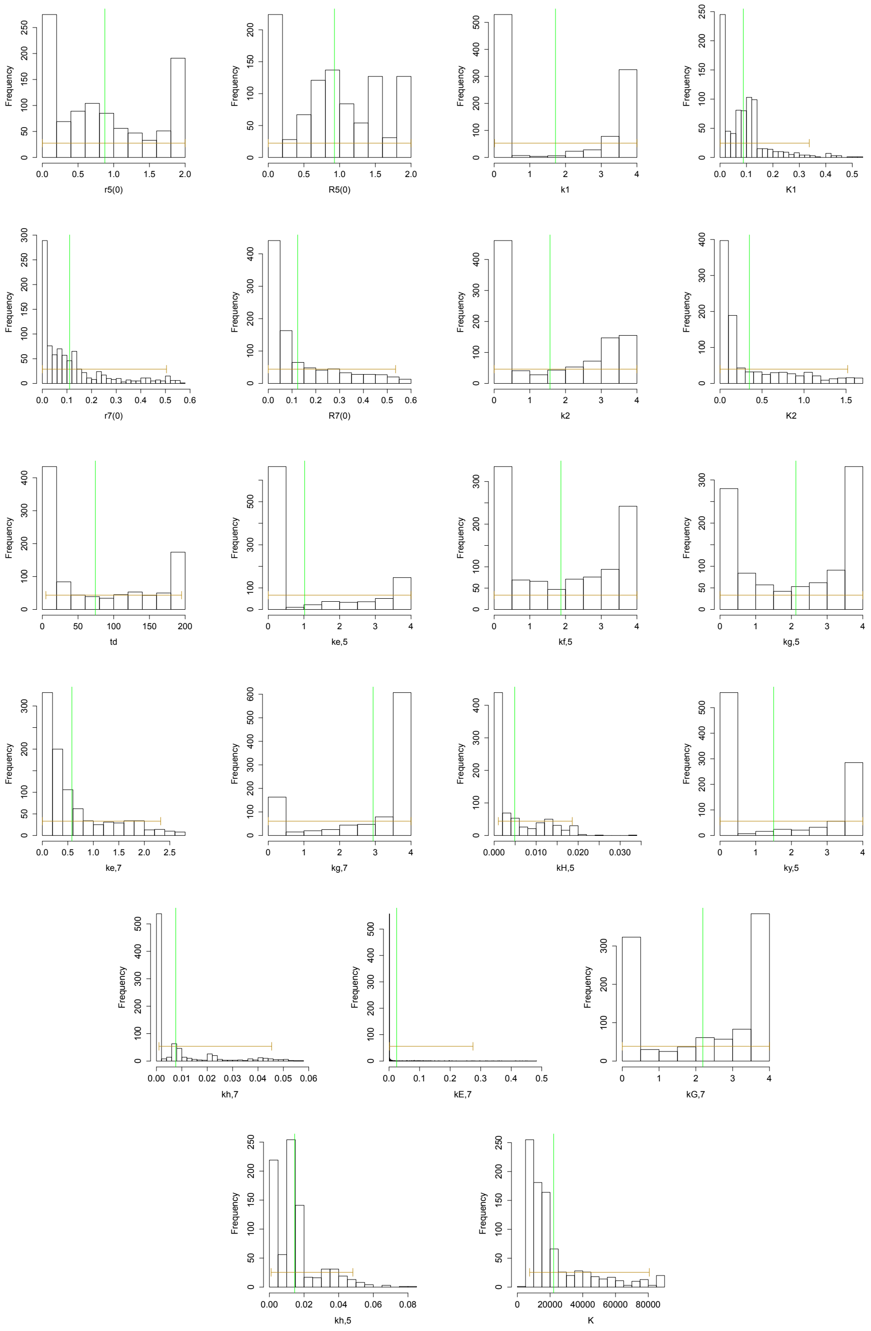

Figure 12: Histograms of the estimated parameter values of the top-ranked model from the IP group obtained using the bootstrap method. In each histogram, the yellow horizontal line represents the 95% confidence interval and the green vertical line represents the mean of the outliers-filtered sample. The width of the bins is calculated according to the Freedman-Diaconis rule.
